# Regulation of Hematopoietic Cell Development and Function Through Phosphoinositides

**DOI:** 10.3389/fimmu.2018.00931

**Published:** 2018-05-04

**Authors:** Mila Elich, Karsten Sauer

**Affiliations:** ^1^Department of Cell and Molecular Biology, The Scripps Research Institute, La Jolla, CA, United States; ^2^Department of Immunology and Microbiology, The Scripps Research Institute, La Jolla, CA, United States; ^3^Oncology R&D, Pfizer Worldwide R&D, San Diego, CA, United States

**Keywords:** phosphoinositide 3 kinase, AKT, SH2 domain-containing inositol-5-phosphatase, phosphatase and tensin homolog, ORAI, ITPKB/IP_3_-3KB/IP3KB, ITPKC/IP_3_-3KC/IP3KC, Kawasaki disease

## Abstract

One of the most paramount receptor-induced signal transduction mechanisms in hematopoietic cells is production of the lipid second messenger phosphatidylinositol(3,4,5)trisphosphate (PIP_3_) by class I phosphoinositide 3 kinases (PI3K). Defective PIP_3_ signaling impairs almost every aspect of hematopoiesis, including T cell development and function. Limiting PIP_3_ signaling is particularly important, because excessive PIP_3_ function in lymphocytes can transform them and cause blood cancers. Here, we review the key functions of PIP_3_ and related phosphoinositides in hematopoietic cells, with a special focus on those mechanisms dampening PIP_3_ production, turnover, or function. Recent studies have shown that beyond “canonical” turnover by the PIP_3_ phosphatases and tumor suppressors phosphatase and tensin homolog (PTEN) and SH2 domain-containing inositol-5-phosphatase-1 (SHIP-1/2), PIP_3_ function in hematopoietic cells can also be dampened through antagonism with the soluble PIP_3_ analogs inositol(1,3,4,5)tetrakisphosphate (IP_4_) and inositol-heptakisphosphate (IP_7_). Other evidence suggests that IP_4_ can promote PIP_3_ function in thymocytes. Moreover, IP_4_ or the kinases producing it limit store-operated Ca^2+^ entry through Orai channels in B cells, T cells, and neutrophils to control cell survival and function. We discuss current models for how soluble inositol phosphates can have such diverse functions and can govern as distinct processes as hematopoietic stem cell homeostasis, neutrophil macrophage and NK cell function, and development and function of B cells and T cells. Finally, we will review the pathological consequences of dysregulated IP_4_ activity in immune cells and highlight contributions of impaired inositol phosphate functions in disorders such as Kawasaki disease, common variable immunodeficiency, or blood cancer.

## Introduction

**Figure 1:**
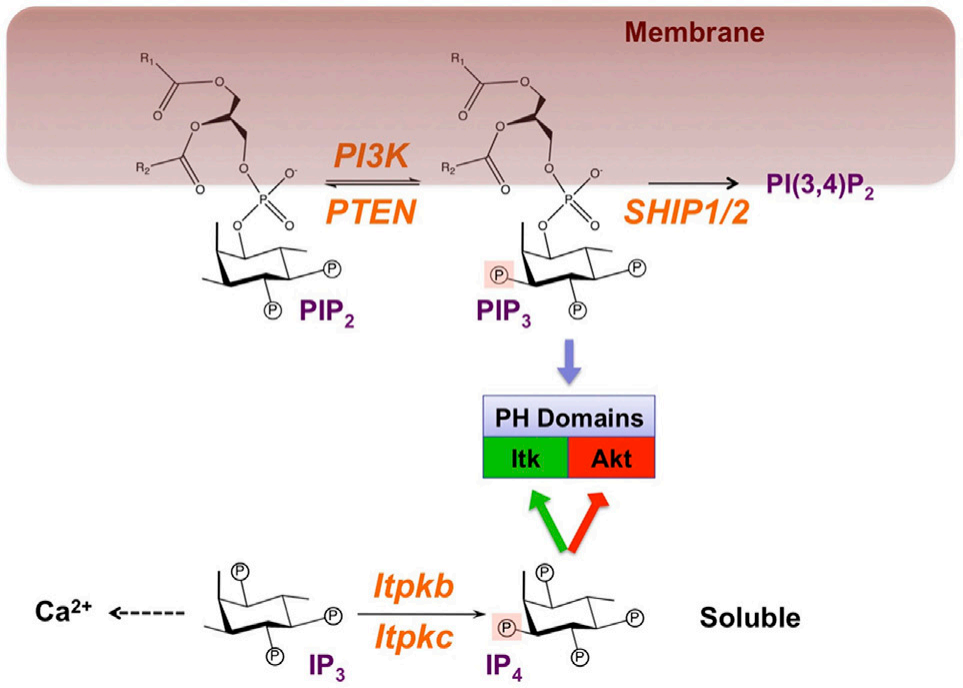
Symmetric signaling by phosphoinositide 3 kinase (PI3K) and Itpkb controls effector recruitment through the analogous but phase-separated pleckstrin homology (PH) domain ligands PIP_3_ and IP_4_. Engagement of antigen receptors activates PI3K, which phosphorylates the membrane-lipid phosphatidylinositol(4,5) bisphosphate (PIP_2_) on the 3-position of its cytoplasm-exposed inositol ring to generate phosphatidylinositol(3,4,5) trisphosphate (PIP_3_). Alternatively but not shown to emphasize the PI3K/Itpkb symmetry, phospholipase-Cγ1 (PLCγ1) can hydrolyze PIP_2_ into the second messengers diacylglycerol (DAG) and soluble inositol(1,4,5) trisphosphate (IP_3_). Canonically, PIP_3_ accumulation is limited through its removal by two families of phospholipid phosphatases: Phosphatase and tensin homolog (PTEN) which reverses the PI3K reaction, and SH2 domain-containing inositol polyphosphate-5-phosphatases (SHIP-1/2) which convert PIP_3_ into phospatidylinositol(3,4) bisphosphate [PI(3,4)P_2_]. Mainly through their IP headgroups, PIP_2_, PIP_3_, and PI(3,4)P_2_ can bind to PH and other domains in signaling proteins such as Itk and Akt, and recruit them to membranes. IP_3_ mobilizes Ca^2+^ but can also be phosphorylated at its 3-position into Inositol(1,3,4,5) tetrakisphosphate (IP_4_) by IP_3_ 3-kinases (Itpka/b/c and inositol-phosphate-multikinase) (8, 19). Because it resembles the PIP_3_ headgroup, IP_4_ can also bind to certain PIP_3_-binding PH and other domains and promote (green) or inhibit (red) PIP_3_ binding. In CD4^+^CD8^+^ thymocytes, IP_4_ promotes PIP_3_ binding to the Itk/Tec PH domains to establish a feedback loop of PLCγ1 activation (20, 21). In neutrophils, NK cells, CD4^−^CD8^−^ thymocytes undergoing β-selection and in hematopoietic stem cells (HSC), IP_4_ competition with PIP_3_ or PI(3,4)P_2_ for binding to its PH domain may limit Akt membrane recruitment and activation (22–27). IP_4_ can also inhibit RASA3/GAP1^IP4BP^-binding to PI(4,5)P_2_ or PIP_3_ (28, 29). Whether this occurs in immunocytes remains unknown. R_1_, R_2_, fatty acid side-chains. Circled P, phosphate moiety. Orange, enzymes with demonstrated physiological relevance in immunocytes.

In one of the most paramount receptor-induced signal-transduction mechanisms, class I phosphoinositide 3 kinases (PI3K) phosphorylate the membrane-lipid phosphatidylinositol(4,5)bisphosphate [PI(4,5)P_2_, hereafter PIP_2_] into the lipid second messenger phosphatidylinositol(3,4,5)trisphosphate [PI(3,4,5)P_3_, hereafter PIP_3_, Figure 1]. By binding to their pleckstrin homology (PH) or certain other domains, PIP_3_ recruits key signaling effectors to cellular membranes, enabling their incorporation into signaling complexes and activation (1). Important examples in lymphocytes include the tyrosine kinase expressed in hepatocellular carcinoma (Tec)-family protein tyrosine kinases (TFK) IL-2-inducible T-cell kinase (Itk), Tec, and Bruton’s tyrosine kinase (Btk). TFK have essential functions in antigen–receptor signaling (2, 3). PIP_3_ also recruits the kinase Akt, a key promoter of cell survival, proliferation, differentiation, and activation. PI3K/Akt dysregulation contributes to immunodeficiencies, autoimmune diseases, allergies, and cancer (4–11). In this review, we discuss how immune cells use inositolphosphates (IPs) as soluble analogs of PIP3 and other phosphoinositides to control the functions of their lipid counterparts and other important cellular processes (Table 1).

**Table 1:** Table of content.

1. Introduction
2. Non-canonical antagonism by IP_4_ prevents excessive PI3K signaling in hematopoietic cells
* 2.1. IP_4_ limits neutrophil function*
* 2.2. Itpkb limits myelopoiesis from GMP*
* 2.3. Itpkb dampens NK cell function*
* 2.4. Itpkb is required for HSC quiescence and longevity*
* 2.5. Itpkb is required for thymocyte β-selection by dampening Akt/mTORC1 function*
3. IP_7_ may antagonize PI3K in neutrophils
4. IP_4_ may promote PI3K signaling to enable thymocyte positive selection
5. IP_4_ dampens store-operated Ca^2+^ entry (SOCE) in immunocytes to promote survival and prevent inflammatory disease
* 5.1. Itpkb is required for T cell viability and function*
* 5.2. Itpkc dampens Ca^2+^ mobilization in immune cells to prevent inflammatory disease*
* 5.3. Itpkb dampens SOCE in B cells*
* 5.4. Itpkb dampens SOCE in neutrophils*
6. Does Itpkb inhibition have therapeutic potential in human diseases?
7. Conclusion and Perspectives

**Figure 2:**
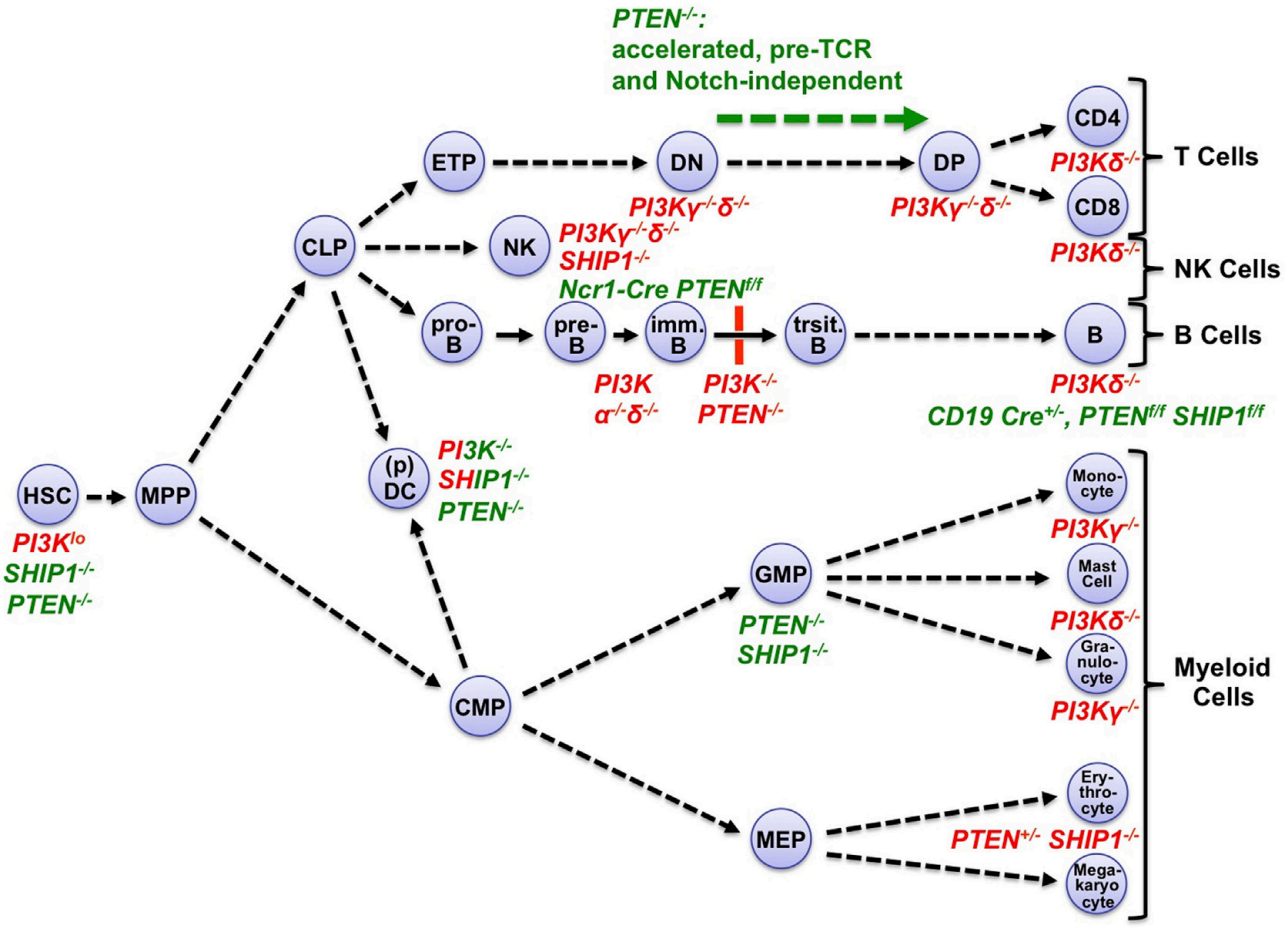
Phosphoinositide 3-kinase (PI3K) loss-of-function or gain-of-function affects multiple stages of hematopoietic development, and mature hematopoietic cells. Hematopoiesis originates from quiescent, long-lived, and pluripotent hematopoietic stem cells (HSC) which reside in BM (BM) niches with low-metabolic and cell cycle activity (26, 30). After occasional division and activation, HSC daughter cells can differentiate through multiple hematopoietic progenitor cell stages including multipotent progenitors (MPP) into lymphoid or myeloid lineages. During lymphopoiesis, MPP-derived common lymphoid progenitors (CLP) give rise to the T cell, NK cell, and B cell lineages. CLP can also generate subsets of dendritic cells (DC), in particular plasmacytoid DC (pDC). CLPs initiate the B/T cell lineages through early thymic progenitors (ETP) and pro-B cells, respectively. ETPs develop through CD4^−^CD8^−^ (DN) and CD4^+^CD8^+^ (DP) stages into mature T cells. In the bone marrow, pro-B cells develop *via* pre-B cells into immature B cells. These translocate into the spleen to mature through transitional stages into mature B cells. In myelopoiesis, MPP-derived common myeloid progenitors (CMP) give rise to granulocyte–monocyte progenitors (GMP) which in turn generate granulocytes, monocytes, and mast cells. Alternatively, CMP can give rise to megakaryocyte–erythrocyte progenitors (MEP), which in turn generate megakaryocytes and erythrocytes. CMP can also generate common DC precursors, which in turn generate most DC subsets (31). The map indicates major hematopoietic progenitors and mature cell types that are negatively (red font) or positively (green font) affected in mice deficient for the indicated *PI3K* isoforms, *SHIP-1*, or *PTEN* (4–9, 26, 30–34). Mixed red–green font indicates complex phenotypes with activation and inactivation components. Immune cells express multiple class I PI3K isoforms. Among those, mature T cell, B cell, NK cell, and mast cell functions or chemotaxis are particularly dependent on the protein tyrosine kinase-dependent receptor-activated PI3Kδ with contributions by the GPCR-activated PI3Kγ (32, 33). Monocyte/macrophage and granulocyte chemotaxis is critically dependent on PI3Kγ, with contributions by PI3Kδ and, in macrophages and neutrophilic granulocytes, PI3Kβ (33, 35). DC require PI3Kγ and δ for various aspects of their function (33). For detailed recent reviews of PI3K isoform functions in hematopoietic cells, see Ref. (32, 33).

Evidenced by the phenotypes of mice lacking the hematopoietically enriched PI3Kγ and PI3Kδ, reduced PIP_3_ signaling impairs most aspects of hematopoiesis, including hematopoietic stem cell (HSC) homeostasis and the development or function of T, B, and NK cells, myeloid mast cells, monocytes, granulocytes, and erythrocytes (4–9) (Figure 2). Limiting PIP_3_ signaling is particularly important, because excessive PIP_3_ function not only oppositely affects many of the same hematopoietic processes but can also transform lymphocytes and cause blood cancers. This is shown by the phenotypes of mice lacking the phosphoinositide-phosphatases phosphatase and tensin homolog (PTEN) or SH2 domain-containing inositol-5-phosphatase-1 (SHIP-1), which canonically limit PIP_3_ function by dephosphorylating it back into PIP_2_, or into PI(3,4)P_2_, respectively (8) (Figure 1). Moreover, PTEN is a pivotal tumor suppressor, and SHIP-1 and PTEN cooperatively suppress B cell lymphomagenesis (12). Besides SHIP-1, hematopoietic cells also express the closely related SHIP-2 (13–15). SHIP-2 dampens immunoglobulin-receptor signaling in macrophages and mast cells (16, 17). Its functions in lymphocytes remain to be elucidated. Highlighting the translational importance of preventing PIP_3_ hyperactivity in hematopoietic cells, the PI3Kδ inhibitor Idelalisib is approved for treating relapsed chronic lymphocytic leukemia (CLL), follicular B-cell non-Hodgkin lymphoma, and small lymphocytic lymphoma (18). Oncogenic PI3K mutations in 50% of human cancers, PTEN status as the second most-often mutated tumor suppressor gene in human cancers, and multiple efforts to therapeutically inhibit PI3K signaling for cancer, metabolic, and immune diseases further illustrate the broad therapeutic importance of preventing PIP_3_ hyperactivity (10, 11).

Adding a non-canonical perspective to the mechanisms controlling PI3K function, we and others found that PIP_3_ activity in hematopoietic cells can also be dampened through antagonism with the soluble PIP_3_-analogs inositol(1,3,4,5)tetrakisphosphate (IP_4_, Figure 1) and inositol-heptakisphosphate, also called diphosphoinositol-pentakisphosphate (hereafter IP_7_) (22–27). Because IP_4_ is identical to the cytoplasm-exposed, PH domain-binding PIP_3_ headgroup, IP_4_ and PIP_3_ can compete for binding to the Akt PH domain. Similarly, IP_7_ can compete with PIP_3_ binding to PH domains (36, 37). Many PH domains bind PIP_3_ and IP_4_ with similar affinities, so IP_4_/PIP_3_ antagonism could be broadly relevant (1, 38). But how many PI3K functions are regulated by IP_4_ and IP_7_ remains a major open question (8, 38). We and others found that in HSC, T cell precursors, NK cells, and neutrophils, IP_4_ dampens PIP_3_ recruitment of Akt; IP_7_ dampens Akt recruitment in neutrophils (22–27). Other evidence suggests that IP_4_ may promote PIP_3_ function in thymocytes undergoing positive selection (20, 21). IP_4_ has additional functions in preventing anergy and death in developing B cells, apoptosis in peripheral T cells, and monocyte hyperactivity that may be unrelated to PI3K (29, 39–44). An emerging common mechanism controlling these different processes is the inhibition of store-operated Ca^2+^ entry (SOCE) through the plasma membrane by IP_4_, its metabolites, or the enzymes producing IP_4_.

**Figure 3:**
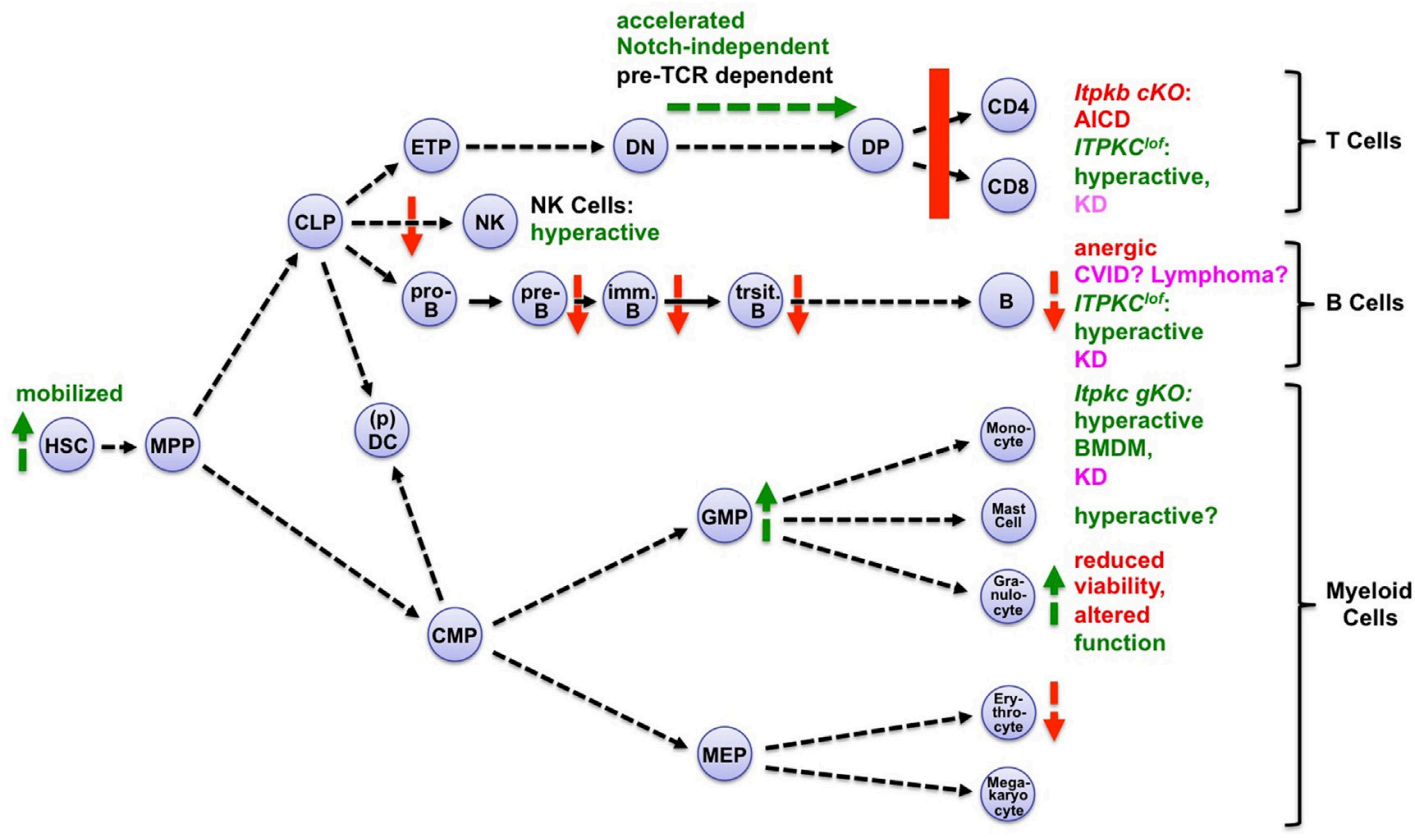
Itpks control multiple aspects of hematopoiesis. Shown are aspects of hematopoiesis affected positively (green symbols and fonts) or negatively (red symbols and fonts) by inactivation of Itpkb or Itpkc. Pink, human diseases associated with loss-of-function (lof) alleles of *ITPKC* (Kawasaki disease, KD) or *ITPKB* (common variable immunodeficiency, CVID). Abbreviations: *cKO*, conditional, *gKO*, germline knockout mice; AICD, activation-induced cell death; BMDM, bone marrow-derived macrophages. Hematopoietic cell stages and pathways are explained in the legend to Figure 2. For more details and references, see text. Mast cells express Itpkb and produce IP_4_ after stimulation (53). Small-molecule Itpk inhibition might augment their activation (54, 55), but the target selectivity of the low-affinity Itpk inhibitors used is unknown and genetic studies are needed. Adapted with permission from Ref. (8).

IP_4_ is produced through phosphorylation of inositol(1,4,5)trisphosphate (IP_3_) by four IP_3_ 3 kinases, three of which belong to the inositol trisphosphate kinase family (Itpka, Itpkb, and Itpkc, Figure 1) (8, 45). Hematopoietic functions of the fourth IP_3_ 3-kinase, inositol phosphate multikinase (IPMK), remain unknown. IP_3_ is an important second messenger that mediates receptor-induced Ca^2+^ mobilization (46). Although many tissues can produce IP_4_, the hematopoietic system has proven particularly useful for elucidating its physiological functions. This may in part reflect a particularly high expression of the best studied IP_3_ 3-kinase, Itpkb, in hematopoietic cells (8, 25). Itpkb is a major producer of IP_4_ in leukocytes, and several studies have used *Itpkb^−/−^* mice to show that IP_4_ deficiency profoundly affects hematopoietic cell development, homeostasis, survival, and function (Figure 3) (20–23, 25, 26, 28, 29, 39, 41–43, 47, 48). Itpkb is also abundant in the brain, which co-expresses Itpka. Itpka is not abundant in immune cells. *Itpka* deficiency caused neurological phenotypes in mice without reported immune defects (49, 50). No significant neurological phenotypes have been reported in *Itpkb^−/−^* mice (8, 45). Loss of the more broadly expressed *Itpkc* in mice hyperactivated macrophages and worsened coronary arteritis in a mouse model for Kawasaki disease (KD) (44), but did not affect other immunocytes as far as studied (44, 47, 51). But reduced ITPKC function in humans may hyperactivate T cells, B cells, and monocytes and promote KD (40, 44). Itpka/b mRNA expression is not affected by immunocyte activation, and Itpk expression profiles are overall comparable between mice and humans (15, 25, 52). Phorbol-12-myristate-13-acetate/ionomycin upregulated ITPKC mRNA in human PBMC and other cells (40).

IP_7_ can be produced in several steps from IP_4_ or other precursors (8, 45). Among the required enzymes, deficiency in *inositol hexakisphosphate kinase-1 (IP6K1)* has unveiled important IP_7_ roles in dampening Akt function in neutrophils (24). IP_4_ and IP_7_ can both be metabolized into various other soluble IPs with unknown functions in lymphocytes, several of which were found in T cells (8, 45, 56).

Below, we review the impact of soluble IPs on hematopoietic cells in detail and discuss current models for how these interesting molecules can have such diverse functions (Table 1). Available data suggest that IP_4_ primarily engages two distinct mechanisms: non-canonical PIP_3_ antagonism to dampen PI3K signaling, and dampening of SOCE to restrict Ca^2+^ mobilization.

## Non-Canonical Antagonism by IP_4_ Prevents Excessive PI3K Signaling in Hematopoietic Cells

*Itpkb* loss in mice causes either hyperactive or loss-of-function (lof) phenotypes in hematopoietic cells (Figure 3). Interestingly, most of the hyperactivation phenotypes appear to result at least in part from Akt hyperactivity due to reduced IP_4_ antagonism with PIP_3_.

### IP_4_ Limits Neutrophil Function

**Figure 4:**
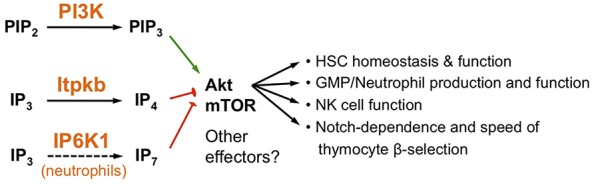
Non-canonical antagonism between phosphoinositide 3-kinase (PI3K) and Itpkb or inositol hexakisphosphate kinase-1 (IP6K1) controls multiple aspects of hematopoiesis. PI3K and Itpkb convert the analogous substrates PIP_2_ and IP_3_ into the analogous products PIP_3_ and IP_4_, respectively. By competing with PIP_3_ for Akt pleckstrin homology domain binding, IP_4_ then dampens PI3K-mediated Akt activation and signaling *via* mammalian or mechanistic target of rapamycin (mTOR). This ensures hematopoietic stem cell (HSC) homeostasis and function, warrants appropriate GMP/neutrophil and NK cell production and function, and establishes the Notch-dependence and kinetics of thymocyte β-selection (22, 23, 25–27). In neutrophils, IP6K1 can also antagonize PI3K activation of Akt by producing the additional PIP_3_ analog IP_7_ (24).

**Figure 5:**
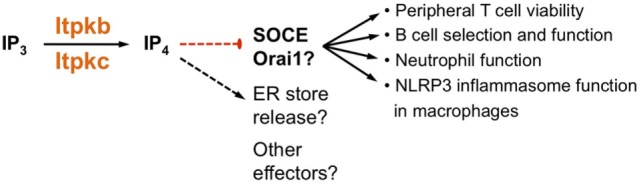
Itpkb controls immune cell biology by dampening store-operated Ca^2+^ entry (SOCE). Several studies suggest that in peripheral T cells, developing and mature B cells, neutrophils and macrophages, Itpkb or Itpkc dampen SOCE through Orai channels (23, 39–42, 44, 59). This may be required for T cell viability, for preventing B cell anergy, and for ensuring neutrophil function. The ability of an exogenously provided cell-permeable IP_4_-ester to reduce SOCE very quickly after administration would be consistent with direct SOCE inhibition through IP_4_ (39, 59). However, variably affected ER store release and previously published, complicated roles for Itpks, IP_3_, IP_4_, and IP_4_ metabolites in controlling Ca^2+^ mobilization in mammalian cells could point to alternate mechanisms and possible other effectors (8, 45, 60, 61). For more detailed discussions, see text.

The intriguing functions of Itpkb and IP_4_ as physiological antagonists of PI3K and PIP_3_ upstream of Akt were first described when the Luo and Schurmans labs characterized Akt gain-of-function phenotypes in *Itpkb^−/−^* neutrophilic granulocytes, an important component of the innate immune system (Figures 1 and 4) (57). Among Itpks, neutrophils mainly express Itpkb (8, 57). Stimulation with chemoattractants such as *N*-formyl-methionyl-leucyl-phenylalanine (fMLP) induced IP_3_ 3-kinase activity and IP_4_ accumulation in neutrophils (23, 57, 58). Upon stimulation with fMLP or the complement factor C5a, bone marrow (BM)-derived neutrophils (BMN) from *Itpkb^−/−^* mice showed increased chemotaxis and superoxide production correlated with Akt hyperphosphorylation and actin hyperpolymerization (57). Akt PH domain–GFP fusion proteins co-precipitated IP_4_, IP_5_, and IP_6_. Treatment with cell-permeable IP_4_ had opposite effects to *Itpkb* knockout on neutrophils and inhibited fMLP-induced Akt PH domain membrane recruitment in HL60 promyelocytic leukemia cells. This suggested that Itpkb dampens chemoattractant-induced neutrophil activation, probably by producing IP_4_ which then competes with PIP_3_ or PI(3,4)P_2_ to inhibit Akt membrane recruitment and activation. Although elevated PI3K/Akt signaling promotes neutrophil viability (23), *Itpkb^−/−^* BMN had reduced viability *in vitro* (22). Thus, *Itpkb* loss probably caused additional defects in neutrophils. Indeed, despite initially reported normal fMLP-induced Ca^2+^ responses in *Itpkb^−/−^* neutrophils (22), follow-up work showed decreased Ca^2+^ store-release but enhanced SOCE (Figure 5) (22, 23, 57). It will be interesting to study if defective Ca^2+^ mobilization underlies the reduced viability.

The effects of *Itpkb* loss on *in vivo* neutrophil function were more complicated. In an acute peritonitis model, neutrophil recruitment into inflamed peritoneal cavities was augmented, but clearance of the injected bacteria was normal or slightly decreased even though *in vitro, Itpkb^−/−^* neutrophils killed serum-opsonized bacteria better than wild-type neutrophils (23, 57). The discrepancy likely reflects a reduced content of opsonizing IgG in the serum of *Itpkb^−/−^* mice due to defects in B cell function (29, 39, 41). Indeed, serum from *Itpkb^−/−^* mice facilitated killing of bacteria less efficiently than wild-type serum (23). Taken together, the data suggest that Itpkb limits neutrophil function, but the physiological consequences are complicated by contributions of defects in other immune cells in *germline Itpkb^−/−^* mice, and by diverse effects of Akt hyperactivation, Ca^2+^ dysregulation, and possibly other perturbed Itpkb/IP_4_ functions (8). Phenotypic similarities between *Itpkb^−/−^* and *PTEN^−/−^* mice include Akt hyperactivation, variably elevated migration, lung or peritoneal recruitment, superoxide production, and bacterial killing (62–65). They are consistent with a PI3K-counteracting Itpkb function. Phenotypic discrepancies such as the elevated viability of *PTEN^−/−^* neutrophils, or massive neutrophil organ-infiltration despite strongly impaired *in vitro* polarization and motility in *SHIP^−/−^* mice (62, 66, 67) might be explained by the aforementioned factors, or by distinct *PTEN* or *SHIP* functions that are unaffected by IP_4_ (25, 26). For example, *SHIP-1* loss increases PIP_3_ levels but may also reduce production of its PH domain-binding product PI(3,4)P_2_, or perturb SHIP-1 scaffolding functions and protein interactions mediated by its adaptor domains (68). *PTEN*-loss causes PIP_3_ accumulation but may also reduce the levels of its product PI(4,5)P_2_, a PLC-substrate and protein ligand (69). IP_4_ can serve as a substrate for PTEN and SHIP-1 *in vitro* (8). Thus, the phenotypes of *SHIP-1^−/−^* and *PTEN^−/−^* mice might involve IP_4_ accumulation, which could limit the PI3K hyperactivation caused by loss of the PIP_3_ phosphatases. Moreover, PIP_3_ controls multiple effectors beyond Akt that can be differentially impacted by IP_4_, Itpks can have IP_4_-unrelated functions such as actin-bundling or removing IP_3_, and Itpkb can control different effectors depending on cell type and context. We discuss these possibilities in detail in Section “Conclusion and Perspectives.”

### Itpkb Limits Myelopoiesis From GMP

Beyond neutrophil hyperactivation, *Itpkb^−/−^* mice also showed increased neutrophil production and peripheral blood numbers. This was associated with increased granulocyte–monocyte progenitor (GMP) proliferation and expansion and suggests that Itpkb restricts myeloid differentiation (22) (Figures 3 and 4). Hematopoietic progenitor cell-enriched BM cells from *Itpkb^−/−^* mice showed increased phosphorylation (activation) of Akt and its substrate, the cell-cycle inhibitor p21^Cip1^. Phosphorylation by Akt decreases cell cycle inhibition by p21^Cip1^, suggesting that Akt hyperactivation promotes GMP expansion by inhibiting p21^Cip1^. Consistent with this view, Akt is essential for myelopoiesis and can promote neutrophil and monocyte development (5, 26). While not formally proven, it is tempting to draw on the recently published HSC regulation by IP_4_ (26) and speculate that Itpkb limits GMP expansion and neutrophil production through IP_4_ antagonism with PIP_3_ for Akt PH domain binding and recruitment (Figure 4). To confirm this, conditional *Itpkb* disruption in GMP and phenotype-rescue studies with cell-permeable IP_4_ or Akt inhibitors will be important. Such studies can also rule out that the GMP phenotype results in part from the earlier HSC mobilization, or indirectly from the defective hematopoiesis and lymphopenia in *Itpkb^−/−^* mice (26).

### Itpkb Dampens NK Cell Function

Other innate immunocytes highly impacted by intrinsic Itpkb loss are NK cells. These recognize and then kill virus-infected or cancer cells through imbalanced signaling by activating (aNKR) and inhibitory (iNKR) NK cell receptors [references in Ref. (25)]. iNKR engagement prevents inappropriate NK cell attack of normal body cells (70, 71). Virus infection or malignant transformation often downregulate iNKR ligands or upregulate aNKR ligands on target cells. The result is NK cell activation, the release of cytolytic granules, and secretion of pro-inflammatory cytokines and chemokines such as IFNγ. All aNKRs ultimately activate PI3K and/or phospholipase-Cγ (PLCγ). PI3K inactivation impairs NK cell maturation, IFNγ production, and cytotoxicity (1, 72–74).

aNKR ligation induced IP_4_ production in NK cells (75). Given the importance of PI3K in NK cells and the ability of IP_4_ to antagonize it, we assessed how loss of Itpkb and thus IP_4_ affects NK cells in *Itpkb^−/−^* mice. We found that *Itpkb* loss cell-autonomously elicited a more immature NKR repertoire and a reduced fraction of CD11b^+^CD27^−^ most mature, long-lived NK cells compared with wild-type mice (25). *Itpkb* loss also increased the proportion of NK cells responding to NKR engagement and augmented effector functions, including IFNγ production, cytolytic granule release, and *in vivo* clearance of target cells lacking iNKR-engaging major histocompatibility complex I molecules. This was, at least in part, caused by defective dampening of PI3K-mediated Akt activation by IP_4_, because *Itpkb^−/−^* NK cells contained hyperactive Akt and treatment with cell-permeable IP_4_ or selective Akt- or PI3K inhibitors reversed both their Akt hyperactivation and hyperdegranulation (25). These data suggest that IP_4_ cell intrinsically promotes NK cell terminal maturation and acquisition of a mature NKR repertoire, but limits mature NK cell effector functions, in part by dampening Akt activity. Thus, non-canonical antagonism of PIP_3_ and IP_4_ is part of the important mechanisms preventing NK cell hyperactivity (Figure 4). Their limited understanding is a barrier to the development of safe and efficacious NK cell immunotherapies for cancer and virus infections (76, 77). In the future, it will be interesting to study possible IP_4_ roles in NK cell tolerance and to determine whether the *Itpkb^−/−^* NK cell phenotype arises exclusively from Akt hyperactivation or involves the deregulation of other NK cell-expressed PIP_3_ effectors, including Tec kinases or the guanine nucleotide exchange factor Vav (25).

Consistent with a PI3K gain-of-function phenotype in *Itpkb^−/−^* mice, loss of the NK cell-expressed PI3Kγ/δ caused an overall opposite phenotype with less CD11b^+^CD27^+^ NK cells, abnormal NKR repertoires, and reduced NKR-mediated IFNγ production and target cell lysis due to impaired NKR signaling and NK cell migration (1, 72–74). One important difference is that Itpkb promotes NK cell maturation but limits effector functions whereas PI3K promotes both processes (72–74). It will be important to elucidate the mechanistic underpinnings of this dichotomy. Among the PI3K-counteracting PIP_3_ phosphatases, *SHIP-1* deficiency caused NKR repertoire changes distinct from those in *Itpkb^−/−^* mice and impaired effector functions including IFNγ secretion despite Akt hyperactivation (74, 78–81). However, the results were complicated by genetic background dependencies and NK cell dependence on both intrinsic and extrinsic SHIP-1 (82). *PTEN* knockdown in human NK cells mildly elevated cytolytic activity; *PTEN* overexpression reduced cytolysis by human and murine NK cells through mechanisms involving impaired immunological synapse formation without altering NK cell development and NKR repertoire in mice (83). However, overexpression artifacts may likely contribute to these differences from *PI3K^−/−^* mice. In another study, conditional *PTEN* deletion in murine NK cells did not strongly affect their maturation and NKR-induced IFNγ production, but caused NK cell hyperproliferation and hyperresponsiveness to the mobilizing chemoattractant S1P along with variable Akt/mammalian or mechanistic target of rapamycin (mTOR) hyperactivation. This resulted in premature BM egress and reduced lymphoid organ and liver, but elevated peripheral blood and lung NK cell numbers (84). Consistent with impaired tissue homing or -retention, *PTEN^−/−^* NK cells had an impaired ability to migrate to distal tumor sites, but cleared blood-borne tumor cells better than wild-type NK cells. The effects of *Itpkb* loss on NK cell migration remain to be elucidated. Based on the *PTEN^−/−^* phenotype and known PI3Kδ requirements for NK cell migration (1, 72–74), it will be interesting to study if reduced tissue homing or -retention contributes to the mildly reduced splenic NK cell numbers in *Itpkb^−/−^* mice (25). The NK cell phenotypic differences between *SHIP^−/−^* or *PTEN^−/−^* and *Itpkb^−/−^* mice could involve the factors discussed above in the neutrophil section, or NK cell-extrinsic contributions whose elimination requires conditional knockouts. Altogether, more detailed mechanistic and genetic studies to better discern the interplay between Itpkb, SHIP, and PTEN in controlling PI3K function in NK cells should prove exciting.

### Itpkb Is Required for HSC Quiescence and Longevity

**Figure 6:**
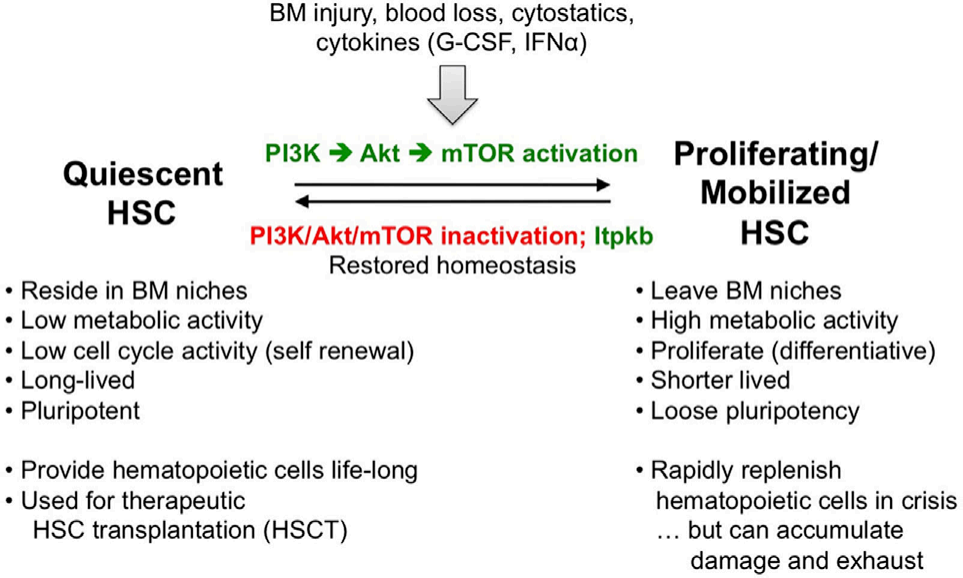
Hematopoietic stem cell (HSC) homeostasis is an exquisitely phosphoinositide 3-kinase (PI3K)-dependent process controlled by Itpkb. To ensure live-long hematopoiesis, HSC reside in BM niches and have low metabolic and cell cycle activity. As a consequence, HSC are long-lived and pluripotent. Stresses including BM injury, blood loss, exposure to cytostatic drugs or cytokines such as G-CSF or type 1 interferons activate and mobilize HSC to leave the BM niches, become metabolically active and proliferate. Some HSC daughter cells then differentiate into hematopoietic progenitors (Figure 2). As a consequence, activated HSC are short-lived and loose their pluripotency. This serves to rapidly replenish hematopoietic cells in a crisis or after HSC transplantation, but persistent HSC activation can lead to HSC damage and exhaustion, ultimately causing BM failure, anemia, immunodeficiencies, or blood cancer (85–87). To prevent this, resolution of HSC-activating stresses normally reverts them into quiescence once the activating stimuli subside. A key mediator of HSC activation that needs to be inactivated for re-entry into quiescence is PI3K signaling *via* Akt and downstream mammalian or mechanistic target of rapamycin (mTOR). In addition, we have identified Itpkb as a promoter of HSC quiescence and homeostasis that acts at least in part by inactivating Akt in HSC (26, 30).

To warrant life-long hematopoiesis, HSC homeostasis must be tightly balanced between quiescence and activation (Figure 6). Persistent activation reduces HSC life span and pluripotency. This can cause immunodeficiencies, anemia, hematopoietic failure, blood cancer, and death (30).

Phosphoinositide 3-kinase is a key regulator of HSC homeostasis. PI3K, Akt, and downstream mTOR complex-1 (mTORC1) are required for HSC self-renewal and function, but also mediate HSC activation and mobilization out of their niches by stresses such as BM injury, blood loss, or treatment with cytostatics or cytokines. This serves to transiently increase hematopoiesis and augment immunocyte or erythrocyte production. Upon resolution of the stress, PI3K inactivation is required for HSC re-entry into quiescence. Excessive PI3K/Akt activity transiently expands HSC, followed by depletion and reduced long-term repopulating capability associated with variable myeloproliferative disease, T-cell acute lymphoblastic (T-ALL) or acute myeloblastic (AML) leukemia (30). Thus, PI3K/Akt activity in HSC needs to be tuned into an appropriate window. Although both PTEN and SHIP have been implicated, the relative importance of HSC-extrinsic vs. -intrinsic PTEN remains controversial, and SHIP-1 may primarily control HSC homeostasis extrinsically by acting in niche cells to prevent production of HSC mobilizing factors and ensure production of HSC-attracting CXCL12 (88).

Because HSC express Itpkb (22, 26), we hypothesized that Itpkb might dampen PI3K/Akt signaling in HSC through PIP_3_/IP_4_ antagonism to ensure their longevity. Supporting this view, young *Itpkb^−/−^* mice accumulated phenotypic HSC with a less quiescent, hyperproliferative phenotype (26). *Itpkb^−/−^* HSC underexpressed genes associated with stemness and quiescence, but overexpressed activation and differentiation-associated genes. They could home into the BM but had reduced persistence and colony-forming activity *in vitro*. *In vivo, Itpkb^−/−^* HSC had a massively reduced competitive long-term repopulating potential. Consistent with severely defective HSC longevity, aging *Itpkb^−/−^* mice lost HSC and other hematopoietic progenitors, and died prematurely with anemia (26).

Increased stem cell factor-mediated Akt/mTORC1 activation in *Itpkb^−/−^* HSC *in vitro* that could be prevented by treatment with cell-permeable IP_4_ or a small-molecule Akt inhibitor, and elevated mTORC1 activity in HSC in *Itpkb^−/−^* mice suggested that Itpkb dampens PI3K/Akt signaling in HSC *via* IP_4_. Moreover, *Itpkb^−/−^* HSC upregulated gene sets associated with Akt/mTORC1 hyperactivity, oxidative phosphorylation, and protein biosynthesis (26). HSC quiescence requires dampened protein biosynthesis and upstream PI3K/mTOR signaling (89). This suggests that the activation of *Itpkb^−/−^* HSC was at least in part caused by exaggerated metabolic activation and protein biosynthesis. Supporting this view, injection of the mTOR inhibitor rapamycin reversed the HSC hyperproliferation in *Itpkb^−/−^* mice (26). We proposed that Itpkb limits cytokine and PI3K/Akt/mTOR signaling in HSC to ensure longevity and prevent BM failure (Figures 4 and 6) (26, 30). Thus, Itpkb is a critical component of the mechanisms which tune PI3K activity in HSC appropriately to balance quiescence and activation.

The transient expansion but later depletion of HSC in *Itpkb^−/−^* mice is reminiscent of the phenotypes resulting from *PTEN* inactivation or expression of dominant-active Akt (90–92). However, T-ALL and AML have not been reported in *Itpkb^−/−^* mice (30). In addition, rapamycin reversed the HSC hyperproliferation in *Itpkb^−/−^* mice but did not rescue their colony-forming activity (26). The reasons remain to be determined, but could include differential effects of Itpkb inactivation, Akt activation, or PTEN loss on PI3K signaling in HSC, or, alternatively, a premature death of *Itpkb^−/−^* mice due to either anemia (26) or infections secondary to immunodeficiency (47) before blood cancer can develop. Itpkb loss might also impair signaling mechanisms required for colony-forming activity or cell transformation that are distinct from PI3K/mTORC1. But, rapamycin also reduced wild-type HSC colony-forming activity (26), and genetic studies suggest mTORC1 requirements for HSC regeneration and function (30). This might explain the difficulty of rescuing *Itpkb^−/−^* HSC function with mTORC1 inhibitors. More detailed biochemical and genetic studies will be needed to fully elucidate how Itpkb controls HSC biology. In particular, conditional *Itpkb* disruption in HSC and large mouse cohorts may help clarify whether *Itpkb* loss can transform blood cells, and whether HSC-extrinsic Itpkb inactivation contributes to the HSC defects in *Itpkb^−/−^* mice (30).

### Itpkb Is Required for Thymocyte β-Selection by Dampening Akt/mTORC1 Function

**Figure 7:**
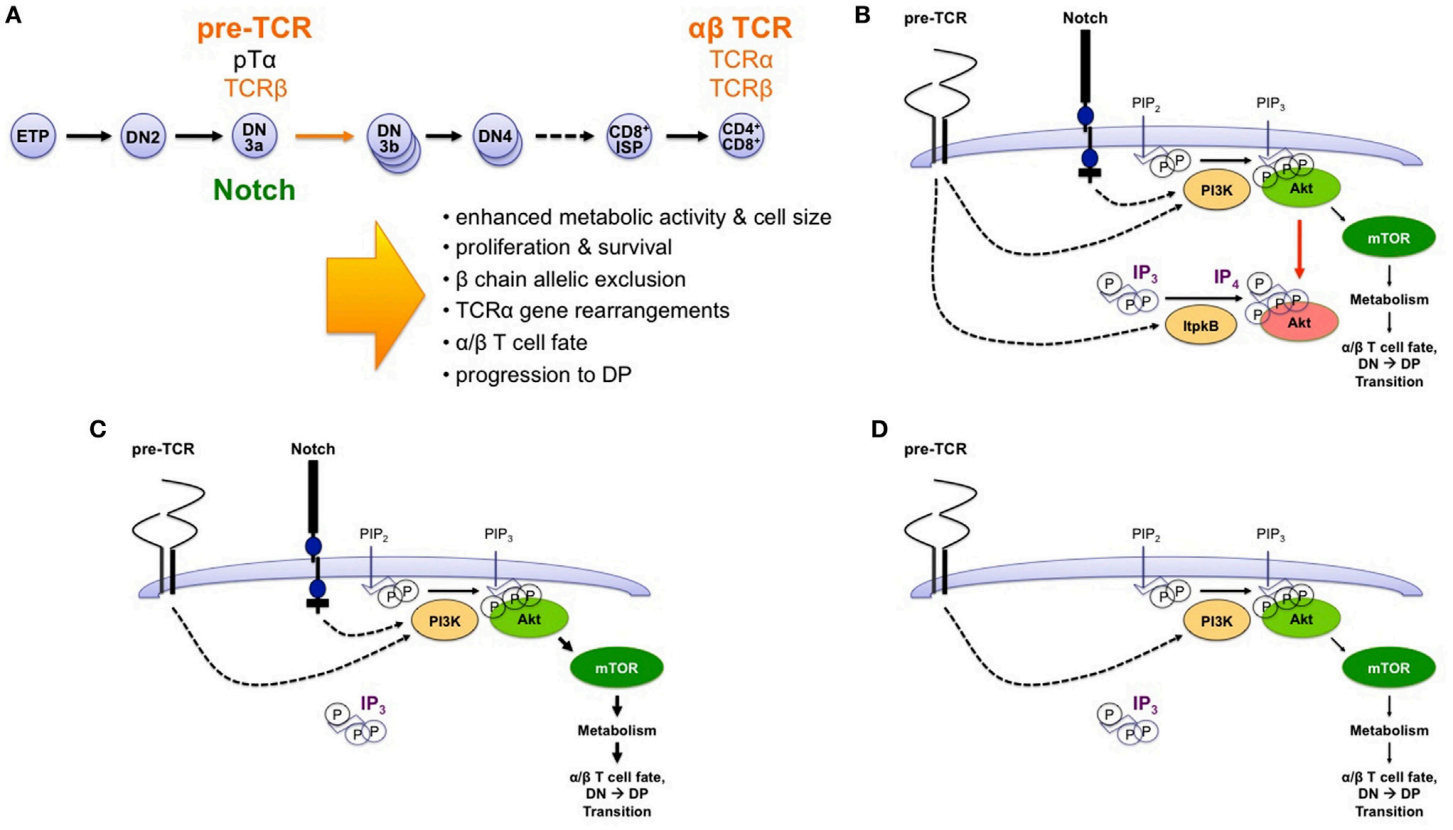
Non-canonical antagonism of phosphoinositide 3-kinase (PI3K) by Itpkb delays thymocyte β-selection and renders it Notch dependent. **(A)** T cells develop in the thymus from HSC and CLP-derived early thymocyte progenitors (ETPs) through several successive CD4^−^CD8^−^ “double-negative” stages (DN2-DN4) and a CD8^+^ immature single-positive (ISP) stage into CD4^+^CD8^+^ double-positive (DP) thymocytes (93, 94). DP cells then undergo positive and negative selection to mature into CD4^+^ or CD8^+^ T cells. At the DN3a stage, expression of a pre-T cell receptor (TCR) composed of an invariant pre-TCRα (pTα) chain and a fully rearranged TCRβ chain triggers metabolic activation, proliferation, survival, β chain allelic exclusion, the initiation of TCRα chain somatic gene rearrangements, acquisition of the α/β T cell fate, and developmental progression to the DP stage. DP thymocytes express a mature TCR composed of fully rearranged α and β chains. The DN3-to-DP transition requires pre-TCR and costimulatory Notch signals. This process is termed β-selection, because it allows only DN3 cells expressing a functional TCRβ chain to survive and mature. **(B)** Based on studies in *Itpkb^−/−^* mice, we recently proposed a model in which pre-TCR and Notch signaling both activate PI3K to produce PIP_3_ in DN3 cells. PIP_3_ then recruits and activates Akt to increase glucose metabolism *via* the Akt/mammalian or mechanistic target of rapamycin (mTOR) pathway. This is required for β-selection. However, pre-TCR signaling also activates Itpkb to produce IP_4_, which competes with PIP_3_ for Akt pleckstrin homology domain binding and limits Akt recruitment and signaling in pre-TCR expressing DN3 cells. By limiting downstream glucose metabolism, this “IP_4_ brake” delays the kinetics of β-selection and renders this process dependent on Notch costimulation (27). **(C)** Without Itpkb, IP_4_ no more dampens Akt activation. In the presence of Notch signals, Akt is now hyperactivated and causes an accelerated DN3-to-DP cell differentiation (indicated by bold arrows). **(D)** In absence of Itpkb, pre-TCR signaling alone sufficiently activates Akt/mTOR to trigger DP cell development without Notch engagement (27).

Recently, we found that beyond innate immunocytes, the paradigm of Itpkb/PI3K antagonism upstream of Akt also applies to adaptive T lymphocytes (27). T cells develop in the thymus from HSC/CLP-derived early thymocyte progenitors (ETPs) through several CD4^−^CD8^−^ “double negative” (DN) stages into CD4^+^CD8^+^ “double positive” (DP) thymocytes which then develop into CD4^+^ and CD8^+^ T cells (93, 94) (Figures 3 and 7A). To generate a diverse T cell repertoire reactive against many pathogens, the T cell receptor (TCR) α and β chain genes somatically rearrange in DN thymocytes. Productive rearrangement of one *TCRβ*-allele causes surface expression of a pre-TCR comprised of TCRβ, invariant pre-TCRα, and signal-transducing CD3 subunits on DN3a cells (95). If a pre-TCR is functional, its ligand-independent signaling triggers DN3 cell metabolic activation, proliferation and survival, allelic exclusion of the second *TCRβ* allele, initiation of *TCRα* gene rearrangements, and differentiation *via* CD8^+^ immature single-positive (ISP) into DP cells (93, 94). This “β-selection” ensures that only DN3 cells expressing a functional TCRβ chain develop further. It is the major cell-fate determining event for αβ T cells. Defective β-selection causes a DN3-block and severe immunodeficiency (4, 95).

β-Selection requires pre-TCR and co-stimulatory Notch signals, which promote DN3 cell metabolism, growth, survival, proliferation, and differentiation. Excessive Notch signaling, however, causes T-ALL. This is augmented by pre-TCR signals (6, 95–100). So, like cytokine signaling in HSC, pre-TCR/Notch signaling in DN3 cells needs to be tuned into an appropriate intensity window.

Both pre-TCR and Notch activate PI3K/Akt (4, 6, 97) (Figure 7B). PI3K/Akt are essential and rate limiting for β-selection by promoting glycolysis, proliferation, survival, and differentiation (6, 101–103). Pinpointing a need to limit PI3K/Akt signaling in DN3 cells for β-selection and its dependence on both pre-TCR and Notch, conditionally *Pten^−/−^* DN cells showed constitutively active Akt and accelerated development to DP cells. They could generate DP cells without pre-TCR or Notch signaling (104–108). But many details about how pre-TCR and Notch crosstalk *via* PI3K are controversial, and it remains unclear why pre-TCR signaling alone is insufficient for β-selection (4, 6, 108). The ability of IP_4_ to antagonize PIP_3_ binding to Akt and documented Itpkb expression and activation by TCR signaling in thymocytes (20, 28, 47, 48) prompted us to explore a role for Itpkb in this process.

We found that *Itpkb^−/−^* DN3 cells were pre-TCR hyperresponsive with Akt/mTOR-hyperactivation and metabolic hyperactivity (27). Mixed BM chimeras and *in vitro* studies showed a DN3 cell-intrinsic Itpkb requirement. *In vitro* and *in vivo, Itpkb^−/−^* DN3 cells showed an accelerated and Notch independent, but pre-TCR dependent differentiation into DP cells with wild-type like proliferation and viability. Pharmacological inhibition of Akt, mTOR, or glucose metabolism restored wild-type developmental kinetics and the Notch dependence of *Itpkb^−/−^* DN3 cells in fetal thymic organ cultures or OP9/OP9-DL1 cell co-cultures. Finally, *Itpkb* codisruption enabled the CD3-induced development of *Rag2^−/−^* DN3 cells into ISP and DP cells in mice injected with a γ-secretase inhibitor which blocks Notch signaling and impaired the maturation of *Itpkb^+/+^Rag2^−/−^* DN cells *in vivo* (27). So, *Itpkb* loss in DN3 cells reduced the Notch dependence of DN thymocyte development to DP cells *in vitro* and *in vivo*.

*Itpkb^−/−^* thymocytes had strongly reduced IP_3_ 3-kinase activity and IP_4_ levels, but normal IP_3_ levels and Ca^2+^ mobilization (20, 28, 47). Based on the IP_4_/PIP_3_ antagonism in other immunocytes, we proposed that pre-TCR induced IP_4_/PIP_3_ antagonism governs β-selection by restricting PI3K/Akt/mTOR signaling and metabolic activation (27). In our model, Itpkb controls pre-TCR/Notch crosstalk through combined restriction of pre-TCR induced and Notch induced PI3K/Akt signaling (Figure 7B). This ensures that Akt is only activated to the extent needed for β-selection and only in an appropriate context: pre-TCR^+^ DN3 cells interacting with Notch-ligand expressing stromal cells in the thymus (93). This prevents premature differentiation. Without Itpkb, excessive Akt signaling accelerates DN3-to-DP development (Figure 7C). In the absence of Notch, Itpkb loss enables pre-TCR signaling alone to sufficiently activate Akt to rescue DN3-to-DP thymocyte development (Figure 7D). Altogether, non-canonical Itpkb antagonism with PI3K both delays thymocyte β-selection and renders it Notch-dependent.

Interestingly, Itpkb has distinct functions from SHIP and PTEN in β-selection. *SHIP-1^−/−^* early thymocytes develop normally (109). Conditionally *Pten^−/−^* DN cells have constitutively active Akt and generate DP cells without pre-TCR or Notch signaling (104–107). And constitutively active Akt allows DN3-to-DP cell development without pre-TCR or Notch-signaling, but not without both (97, 103, 105, 110). Notch may promote β-selection in part by inducing HES1 to repress PTEN, and c-Myc to promote proliferation (107). By contrast, *Itpkb* loss accelerates DN3 cell differentiation without significant effects on proliferation and viability, and overcomes the dependence of β-selection on Notch but not the pre-TCR (27). We hypothesize that the latter reflects the requirement for TCR signals to activate Itpkb and produce IP_4_ (28, 47, 48). Without pre-TCR signals, Itpkb is inactive and its loss has no further effect. *Itpkb* loss might also reduce less essential positive Itpkb roles in pre-TCR signaling, such as promoting Itk activation (20, 111). The PI3K-independent c-Myc induction by Notch (107) should be unaffected by IP_4_. This might explain why *Itpkb* loss overcomes Notch requirements and accelerates DN3 cell differentiation but not proliferation. The surprising lack of increased DN3/DN4 cell viability in *Itpkb^−/−^* mice might reflect differing degrees of Akt/mTOR hyperactivation in *Pten^−/−^*, dominant-active *Akt1-*expressing, and *Itpkb^−/−^* DN3/DN4 cells (27). Finally, the mechanistic differences between Itpkb, PTEN, and Notch regulation of β-selection, and the aforementioned death due to hematopoietic failure or infections (26, 47) might explain why *Itpkb^−/−^* mice do not present the leukemias/lymphomas caused by excessive signaling of Notch, PI3K, or Akt in DN3 cells (6, 95, 98). It will be interesting to study if combined deficiency in *Itpkb* and *PTEN* or *SHIP* causes earlier blood cancer development and increases its incidence.

Wrapping up this section, neutrophils, NK cells, HSC, DN3 thymocytes, and likely GMP provide examples where non-canonical antagonism of Itpkb and PI3K/Akt controls important physiological processes (Figure 4). Thus, IP_4_ antagonism with PIP_3_ is broadly important at least in hematopoietic cells. One major downstream process is metabolism, although other PIP_3_-regulated processes likely contribute depending on cell type and context. Additional roles for PIP_3_-independent functions of IP_4_ and Itpkb cannot be ruled out (8, 45). Consistent with these possibilities, the precise effects of Itpkb, SHIP, or PTEN inactivation in hematopoietic cells often differ. This underscores the distinct importance of Itpks and IP_4_ in controlling hematopoiesis.

## IP_7_ may Antagonize PI3K in Neutrophils

Besides IP_4_, IP_7_ produced by IP6Ks can also compete with PIP_3_ for PH domain binding to dampen PI3K function. This was first shown *in vitro* and in *Dictyostelium discoideum* where *IP6K1* deletion enhanced the membrane translocation of several PH domain-containing proteins and augmented downstream chemotactic signaling (36). A later study showed that through the same mechanism, IP6K1 and IP_7_ dampen Akt function in skeletal muscle, white adipose tissue and liver cells to limit insulin sensitivity (37). In *IP6K1^−/−^* mice, these organs showed elevated Akt/mTOR and reduced GSK3β signaling, resulting in insulin hypersensitivity and resistance to high-fat diet or aging-induced obesity. By contrast, IP_7_ treatment inhibited Akt phosphorylation and activation by PDK1 in a PH domain-dependent manner.

Expanding on these findings, the Luo lab demonstrated that IP_7_ can also dampen PIP_3_ signaling in neutrophils (24) (Figure 4). Neutrophils from *IP6K1^−/−^* mice or human neutrophils treated with a pharmacological IP6K1 inhibitor showed Akt hyperactivation after fMPL treatment, enhanced PIP_3_-mediated membrane recruitment of an ectopically expressed Akt PH domain, elevated phagocytic and bactericidal activity, and augmented Akt-dependent, NADPH-oxidase mediated superoxide production compared to wild-type or untreated neutrophils, respectively. By contrast, overexpression of wild-type but not catalytically inactive IP6K1 in neutrophil-like differentiated HL60 cells (dHL60 cells) caused IP_7_ overproduction and suppressed fMLP-induced Akt activation, membrane recruitment, and downstream superoxide production. And exogenous IP_7_ blocked PI3K-dependent superoxide production in neutrophils. Suggesting physiological relevance of these findings, *IP6K1^−/−^* mice had elevated peritoneal ROS but reduced intraperitoneal bacterial counts in two different acute peritonitis models at early timepoints post-bacterial infection when macrophages and lymphocytes are not yet recruited. This occurred despite attenuated peritoneal neutrophil accumulation, possibly secondary to accelerated bacterial clearance or chemoattractant deactivation by the elevated ROS. Surprisingly, *IP6K1^−/−^* neutrophils showed wild-type like cell adhesion, directionality, migration velocity, and recruitment to the peritoneal cavity upon adoptive transfer and had wild-type like viability *in vitro*, although these processes are PI3K dependent. This somewhat contrasts with the effects of *PTEN* loss in neutrophils and clould reflect different regulation of PI3K by PTEN and IP6K1 in these, non-redundant PI3K dampening by Itpkb in neutrophils, or the surprising but incomplete drop in neutrophil IP_7_ levels after fMLP stimulation (24). Comparing PIP_3_ vs. IP4 vs. IP_7_ amounts and resulting PI3K/Akt activity in neutrophils lacking *PTEN* vs. *IP6K1* vs. Itpkb might further elucidate how differential PIP_3_ antagonism by these enzymes impacts nuances of PI3K signaling.

Despite similarly increased fMPL-induced Akt recruitment and superoxide production, *Itpkb^−/−^* and *IP6K1^−/−^* neutrophils showed several phenotypic differences. In particular, *Itpkb^−/−^* neutrophils had reduced *in vitro* viability, increased chemotaxis and peritoneal recruitment, and normal-to-reduced bacterial clearance *in vivo* (22, 57). By contrast, *IP6K1^−/−^* neutrophils showed unimpaired *in vitro* viability, migration, and peritoneal recruitment but improved bacterial clearance (24). The mechanistic underpinnings of these differences remain to be elucidated. It is tempting to speculate that they include the normal vs. elevated Ca^2+^ mobilization in *IP6K1^−/−^* vs. *Itpkb^−/−^* neutrophils (23, 24, 112), and potential differences in the serum content of opsonizing IgG due to defective B cell functions in *Itpkb^−/−^* mice (29, 39, 41). Whether IP6Ks and IP_7_ have functions in B cells is unknown. Moreover, without conditional knockout mice, differential contributions of possible phenotypes in other immune cells cannot be ruled out but might explain the improved bacterial clearance in *IP6K1^−/−^* mice despite attenuated neutrophil peritoneal accumulation (24). Partial redundancy between IP6K1 and the also neutrophil-expressed IP6K2 is another possibility (24). In addition, IP_7_ can bind multiple proteins including epigenetic regulators, and contrasting with IP_4_ can serve as a non-enzymatic protein phosphorylating agent (113–115). It remains to be elucidated whether these functions play roles in neutrophils. Finally, Ip6k1-mediated inorganic polyphosphate production in platelets promoted alveolar neutrophil accumulation during bacterial pneumonia (116). Distinct features of IP6K1 regulation in neutrophils, and of IP_7_ vs. inorganic polyphosphates, IP_4_ and PIP_3_ may also explain differences between the neutrophil phenotypes of *IP6K1^−/−^, PTEN^−/−^* (63–65), and *SHIP^−/−^* mice (62, 117), summarized above in the Itpkb section and in Ref. (112).

## IP_4_ may Promote PI3K Signaling to Enable Thymocyte Positive Selection

**Figure 8:**
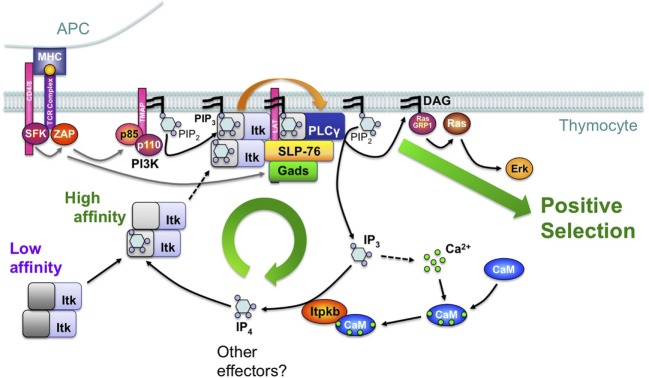
Feedback-activation of Itk/phospholipase-Cγ1 (PLCγ1) by IP_4_ may amplify mild T cell receptor (TCR) signals such that they can trigger thymocyte positive selection. Based on studies of *Itpkb^−/−^* mice and mathematical simulations of different signaling circuitries in DP cells (20, 21, 28, 56), we proposed a simplified model where TCR engagement on DP thymocytes activates proximal protein tyrosine kinases (SFK, ZAP), which then tyrosine-phosphorylate transmembrane adaptors (TMAPs, LAT). Among other events, this activates phosphoinositide 3-kinase (PI3K), which then phosphorylates membrane PIP_2_ into PIP_3_. By binding to their PH domains, PIP_3_ recruits PLCγ1 and its upstream activator, the Tec-family protein tyrosine kinase Itk into the TCR signalosome which also contains LAT and the adaptors SLP-76 and Gads. In this model, Itk is oligomeric with low PH domain affinity for PIP_3_ (dark gray). Therefore, initial Itk recruitment is limited and only triggers low-level PLCγ1 activation. PLCγ1 hydrolyzes PIP_2_ into low amounts of the second messenger diacylglycerol (DAG) and IP_3_. IP_3_ mobilizes Ca^2+^. Ca^2+^ binds calmodulin (CaM) which then binds to and activates Itpkb and calcineurin (CaN) (118). CaN dephosphorylates and activates the transcription factor NFAT (data not shown) (119). Itpkb phosphorylates IP_3_ into IP_4_. IP_4_ binding to one Itk subunit allosterically increases the PH domain affinity of all Itk subunits for PIP_3_ (light gray). This promotes Itk membrane recruitment, causing full PLCγ1 activation and sufficient DAG-production to activate Ras/Erk and trigger thymocyte positive selection. Itpkb loss perturbs this feedback activation through decreased IP_4_ production and Itk recruitment. As a result, insufficient DAG production impairs Ras/Erk activation and positive selection, causing a block of thymocyte development at the DP stage. Itpkb and IP_4_ may have additional functions in DP thymocytes, which also express additional IP_3_ 3-kinases whose roles in positive/negative selection remain unclear (28, 47). For a detailed discussion, see text [adapted with permission from Ref. (8)].

The first hematopoietic defect in *Itpkb^−/−^* mice reported independently by the Schurmans/Erneux group and us was a severe T cell deficiency resulting from blocked thymocyte development at the DP stage (28, 47) (Figure 3). Studying the underlying molecular defect, we found evidence that IP_4_ may promote the PIP_3_-mediated membrane recruitment and activation of Itk downstream of the TCR by acting as a soluble PIP_3_ analog that binds the Itk PH domain and promotes PIP_3_ binding (20) (Figure 8). This was the first demonstration that IP_4_ has an important *in vivo* function and can act as a physiologically relevant PIP_3_ analog, and that Itpkb controls PI3K function *in vivo*.

In DP cells, TCR ligand-sensitivity is assessed through interactions with self-peptide/MHC complexes on thymic stromal cells. Insufficient TCR signals cause thymocyte death by neglect. Adequately mild signals cause DP cell survival and differentiation into CD4 and CD8 single-positive T cells. This “positive selection” ensures that only T cells with a functional TCR develop. Intermediate TCR signals “agonist-select” regulatory T cells. But excessive TCR signals in DP cells cause activation-induced cell death (AICD). This “negative selection” prevents the maturation of self-reactive T cells which could cause autoimmune diseases (120).

T cell receptor stimulation activates proximal protein tyrosine kinases, which then phosphorylate transmembrane adaptors including LAT. Their phosphotyrosine moieties subsequently bind and recruit downstream effectors including PI3K, Itk, and phospholipase-Cγ1 (PLCγ1) (8, 19). Itk recruitment also requires binding of its PH domain to membrane-PIP_3_ (19) (Figure 8). Itk/PLCγ1 co-recruitment to LAT allows Itk to phosphorylate and activate PLCγ1. PLCγ1 then hydrolyzes membrane PIP_2_ into diacylglycerol (DAG) and soluble IP_3_. DAG recruits PKCs, and RAS-GRP1 to activate Ras/Erk signaling. This is required for positive selection (8). IP_3_ binds to IP_3_ receptors in the ER to mobilize Ca^2+^. Alternatively, Itpkb can convert IP_3_ into IP_4_. In some cells, IP_4_ can also control Ca^2+^ mobilization (19, 45, 61, 121).

In *Itpkb^−/−^* mice, positive selection was severely blocked. Data about negative selection were negative or inconclusive (8, 20, 28, 47). As expected, *Itpkb^−/−^* DP cells showed reduced TCR-induced IP_4_ production. Although Itpkb loss was expected to cause IP_3_ accumulation and *Itpkb^−/−^* peripheral T cells showed elevated Ca^2+^ mobilization (59), *Itpkb^−/−^* DP cells produced normal amounts of IP_3_ and Ca^2+^ (28, 47). The inability of catalytically inactive Itpkb, but ability of exogenous IP_4_ to restore positive selection of *Itpkb^−/−^* DP cells suggested a specific IP_4_ requirement for this pivotal process (8, 20). Biochemical studies then showed that in *Itpkb^−/−^* DP cells, TCR-induced Erk activation was impaired because of defective Itk membrane recruitment and activation. This impaired PLCγ1 activation and DAG production (20, 28). Compensation of reduced IP_3_ turnover *via* Itpkb by reduced PLCγ1-mediated IP_3_-production might explain the normal IP_3_ levels in *Itpkb^−/−^* DP cells (20).

The dual ability of IP_4_ to bind to the Itk PH domain and impair PIP_3_-binding at high, but promote PIP_3_-binding at low, physiological concentrations then suggested that IP_4_ might be required for Itk membrane recruitment and activation by augmenting Itk PH domain binding to PIP_3_ (20). Although the precise mechanism remains to be fully elucidated, the ability of full-length Itk or its PH domain alone to oligomerize is consistent with a model where IP_4_ binding to one Itk-subunit induces allosteric changes in the other Itk-subunits that cooperatively increase the affinity of their PH domains for PIP_3_ (2, 3, 8, 20, 122) (Figure 8).

These data suggest that in DP cells, IP_4_ may establish a positive feedback loop of PLCγ1 activation by Itk that is required for the production of sufficient DAG to activate Ras/Erk and trigger positive selection (8, 20). Ca^2+^ controls signaling by binding to various proteins, including calmodulin (CaM). Ca^2+^/CaM can bind and activate Itpkb (45, 48, 123–126). TCR-induced IP_4_ production requires Itpkb, Ca^2+^, and CaM (8, 28, 47, 56, 127, 128). This and the inability of CaM-nonbinding mutant Itpkb to restore *Itpkb^−/−^* thymocyte maturation (20, 48) suggested that TCR-induced IP_4_ production in DP cells may involve an additional feed-forward loop of Itpkb activation by Ca^2+^/CaM downstream of PLCγ1 (8). We proposed that this combination of feedback- and feedforward-activation loops establishes an IP_4_- and Ca^2+^-dependent signal amplifier that allows mild TCR stimuli to trigger positive selection, but is dispensable for negative selection triggered by strong stimuli (8). This circuitry might also underlie a previously proposed signal splitter that directs selection outcome (129). Supporting this model, strong TCR stimuli rescued DAG production and Erk signaling in *Itpkb^−/−^* thymocytes (20, 28). However, detailed studies in sensitive models (130) will be required to conclusively determine how Itpkb loss affects negative and agonist selection.

Due to difficulties in quantifying Itk interactions with PIP_3_ and IP_4_, in monitoring Itk-oligomerization *in vivo*, and in generating non-oligomerizing Itk mutants, the physiological relevance of Itk oligomerization remains controversial (20, 122, 131–136) and many mechanistic details of how IP_4_ controls Itk remain to be elucidated. Providing conceptual support for the model in Figure 8, computational simulations of various circuitries involving mono- or oligomeric Itk indicated that those models which shared a cooperative-allosteric Itk regulation by IP_4_ involving oligomeric PH domains were most robust against variations of reactant amounts and kinetic rates at the single-cell level (21). Interestingly, some models predicted an additional benefit for Itk inhibition through PIP_3_ antagonism by high doses of IP_4_. Although high-dose IP_4_ can inhibit Itk PH domain binding to PIP_3_, it is unknown whether such high doses can be achieved in DP cells (20). Further exploration of bimodal Itk regulation by IP_4_ and of the physiological relevance of different Itk dimers remain important future research areas (21). Finally, recent evidence that in TCR-stimulated thymocytes, Itpkb is phosphorylated by Erk which is counteracted by the Ca^2+^-activated phosphatase calcineurin suggests complex additional circuitries whose physiological relevance remains to be elucidated (137).

Although Itpkb is pivotal for positive selection, residual Itpk activity and IP_4_ production in *Itpkb^−/−^* DP cells suggest relevance for other IP_3_ 3-kinases and that complete IP_4_ loss could have more severe phenotypes (28, 47). This would be consistent with the broader roles of PI3K and Itk in thymocyte selection (2–5, 111, 138–142). Co-disruption of several IP_3_ 3-kinases in DP cells will be required to address this question. Moreover, it will be important to generate conditionally *Itpkb*-deficient mice and exclude contributions of the defects in HSC (26) and β-selection (27) to the DP thymocyte defects in *germline Itpkb^−/−^* mice. Finally, differences in the selection phenotypes of *Itpkb^−/−^* and *Itk^−/−^* mice point toward possible roles for other IP_4_ targets, or for Itpkb interactions with actin (8, 45, 143, 144). Thus, deeper mechanistic studies should yield important additional insight.

## IP_4_ Dampens Store-Operated Ca^2+^ Entry (SOCE) in Immunocytes to Promote Survival and Prevent Inflammatory Disease

### Itpkb Is Required for T Cell Viability and Function

Peripheral T cells express all three Itpks. TCR stimulation induced IP_3_ 3-kinase activity and IP_4_ production in Jurkat T cells (56, 127). To elucidate the functions of Itpkb and IP_4_ in peripheral T cells, two studies have used different approaches aimed to leave T cell development intact. The Cooke group combined studies of mice with tamoxifen-induced conditional *Itpkb* disruption (*Itpkb cKO*) with studies of the effects of a specific and selective, orally bioavailable pan-Itpk small-molecule inhibitor, GNF362 (59). Tamoxifen treatment of *Itpkb cKO* mice caused a mild defect in positive selection that contrasts with the severe block in germline *Itpkb^−/−^* (*Itpkb gKO*) mice (20, 28, 47). Indeed, *Itpkb cKO* mice had control-like numbers of splenic B and T cells. Compared to *Cre^+^unfloxed* controls, *Itpkb cKO* T cells had normal basal viability but underwent AICD after TCR stimulation (59). Intact cytokine production may suggest that this is their primary defect, consistent with rescued T cell viability and proliferation by FasL blockade. Supporting T cell malfunction, *Itpkb cKO* mice failed to generate antibody responses to T cell-dependent but not -independent antigens.

Following IP_3_-mediated Ca^2+^ release from ER stores, STIM1 proteins in the ER sense the resulting Ca^2+^ depletion, translocate close to the plasma membrane and activate Orai channels which mediate SOCE. This is essential for T cell activation (119). Interestingly, *Itpkb cKO* T cells showed enhanced SOCE, and treatment with high doses of cell-permeable IP_4_ rapidly inhibited SOCE in HEK293 cells overexpressing STIM1 and ORAI (59). The opposing effects of Itpkb loss and IP_4_ treatment on SOCE might suggest that Itpkb limits SOCE through IP_4_. Consistent with this view, GNF362-treatment blocked IP_4_ production in Jurkat T cells and enhanced TCR-induced SOCE in thymocytes and murine T cells. GNF362 also inhibited T cell proliferation and caused Itpkb-dependent AICD (59). In mice, GNF362 recapitulated the blocked T cell development seen in *Itpkb^−/−^* mice. Consistent with T cell inhibition, GNF362 inhibited joint swelling and secondary antibody responses in a rat antigen-induced arthritis model (59).

While the precise mechanism through which Itpkb and IP_4_ inhibit SOCE in T cells remains to be elucidated, elevated Ca^2+^ mobilization can induce pro-apoptotic genes to mediate AICD, and *Orai1*-deficient T cells are resistant to AICD (145). So, the elevated SOCE in *Itpkb cKO* T cells might explain their AICD (59). However, phenotype rescue through pharmacologic or genetic prevention of the SOCE elevation in Itpkb-inactivated T cells will be required to prove this. Otherwise, it remains possible that the AICD of Itpkb-inactivated T cells results at least in part from a hypersensitivity to TCR stimulation or generally increased TCR signals. Given the hyper-responsiveness of *Itpkb^−/−^* HSC, DN thymocytes, NK cells, and neutrophils to stimulation, this remains a possibility worth testing. Then again, based on the defective Itk/PLCγ1 activation in *Itpkb^−/−^* DP cells (20) and the Akt/mTOR hyperactivation in *Itpkb^−/−^* DN thymocytes (27), *Itpkb*-deficient peripheral T cells could have complex additional defects with loss-of-function and gain-of-function components that remain to be explored.

In an independent study, transient transgenic *Itpkb* expression partially rescued thymocyte development in another line of *Itpkb gKO* mice (43). These but not mice transiently expressing *catalytically inactive Itpkb* showed partially restored SP thymocytes. They also had low numbers of peripheral T cells with an activated/memory phenotype but decreased TCR-induced proliferation and survival, and increased cytokine secretion compared to wild-type mice. TCR-induced Ca^2+^ mobilization was not significantly altered.

The reduced proliferation and survival of *Itpkb* transgene-rescued *Itpkb gKO* T cells are consistent with the *Itpkb cKO* or GNF362-treated T cell phenotypes (59). However, the activated/memory phenotype and cytokine hypersecretion contrast with those. Possible reasons could be homeostatic expansion of the few transgene-rescued T cells, or confounding effects of infections. Moreover, transgenic *Itpkb* was expressed from the Lck proximal promoter which transiently expresses transgenes in DN and DP thymocytes but not in HSC (146). So, unrescued HSC defects in *Itpkb gKO* mice (26), the super-physiological amount of transgenic Itpkb in the rescued thymocytes (43), the incomplete rescue of thymocyte development, or low residual transgene expression in peripheral T cells could all possibly affect T cell phenotypes.

Wrapping up, both studies suggest that Itpkb and IP_4_ have critical functions in ensuring the survival and function of activated peripheral T cells (Figure 5). The underlying mechanism may involve IP_4_ dampening of SOCE, but the molecular details remain to be explored and other possibilities have not been ruled out. Clearly, further studies of how Itpkb controls T cell function should prove exciting.

### Itpkc Dampens Ca^2+^ Mobilization in Immune Cells to Prevent Inflammatory Disease

Despite its broad expression, studies in *Itpkc^−/−^* mice have not yet unveiled lymphocyte phenotypes, and co-disruption of *Itpkb* and *Itpkc* did not worsen the thymocyte defects in *Itpkb^−/−^* mice. *Itpkc^−/−^* thymocytes showed unaltered IP_3_ 3-kinase activity (47, 51). This argues against major Itpkc roles in adaptive immune responses in mice. By contrast, human population genetics suggest that ITPKC may limit Ca^2+^ mobilization in, and function of human T cells (Figure 5). In a seminal study (40), Onouchi et al. found an interesting association of a human *ITPKC* allele that reduced *ITPKC* mRNA splicing efficiency and abundance (*ITPKC^lof^*) with increased susceptibility to KD, a multisystem inflammatory vasculitis that mainly affects coronary arteries (147). KD is the leading cause of childhood-acquired heart disease in developed countries (40). Several subsequent studies confirmed the *ITPKC^lof^* genetic association, although others found no evidence for it, likely due to different subject cohorts with unknown confounding genetic and environmental influences (148).

Acute phase KD patients showed T cell infiltration into the coronary artery wall and IL-2 overproduction, suggesting T cell hyperactivation. PMA/ionomycin treatment upregulated ITPKC mRNA levels in human T cells, and *ITPKC* overexpression decreased, but *ITPKC* knockdown increased, phytohemagglutinin- and PMA-induced NFAT activation and IL-2 mRNA expression in Jurkat cells. This suggests that ITPKC inhibits human T cell activation upstream of the Ca^2+^-activated transcription factor NFAT (40, 149). Another important advance in our understanding of how ITPKC controls KD was provided by the recent finding that Itpkc limits Ca^2+^ mobilization in myeloid cells to restrict activation of the NLRP3 inflammasome (44). Compared to wild-type controls, bone marrow-derived macrophages from *Itpkc^−/−^* mice had elevated basal and ionomycin-induced Ca^2+^ levels and NLRP3 expression. They responded with NLRP3 hyper-induction and excessive release of pro-inflammatory IL-1β to *in vitro* activation by LPS/ATP or *Lactobacillus casei* cell wall extract (LCWE). In a LCWE-induced KD model, *Itpkc^−/−^* mice overproduced circulating IL-1β and developed a more severe disease compared to wild-type controls.

Ascribing human relevance to these findings, acute-phase KD patients had higher serum levels of IL-1β, IL-18, and their antagonists IL-1RA and IL-18BP than convalescent and age-matched febrile controls (44). Whole blood from acute-phase KD patients also hyperexpressed a gene signature suggesting NLRP3 activation. Interestingly, EBV-immortalized B cells from KD patients or healthy controls harboring homozygous *ITPKC^lof^* had reduced Itpkc protein levels. They recapitulated the elevated basal and ionomycin-induced Ca^2+^ levels of murine *Itpkc^−/−^* macrophages, showed a more sustained Ca^2+^ mobilization, and overexpressed NLRP3 (44). They also overproduced mitochondrial superoxide, a Ca^2+^-dependent NLRP3-activator. So, Itpkc loss in human B cells associates with Ca^2+^ hypermobilization, which likely triggers superoxide-mediated NLRP3 activation. Acute phase KD patients carrying homozygous *ITPKC^lof^* also showed elevated plasma concentrations and LPS/ATP-stimulated PBMC production of IL-1β and IL-1. This suggests that the NLRP3 hyperactivity caused overproduction of pro-inflammatory cytokines, similar to *Itpkc* loss in mice. Increased resistance to standard IVIG therapy in KD patients carrying *ITPKC^lof^* supports pathological relevance of these effects (44). These observations suggest interesting similarities between KD and recurrent fever syndromes that may reflect causative NLRP3 hyperactivity. They may explain the efficacy of IL-1 blockade in recalcitrant KD and may identify IL-1β, IL-18, and their antagonists as much-needed biomarkers for early diagnosis (44).

Intriguingly, KD may not be the only disease affected by Itpkc. Recent studies found potential associations between *ITPKC* genetic variations and Hirschsprung disease, calcium nephrolithiasis, and cervical squamous cell carcinoma (150–152). Thus, further mechanistic studies of Itpkc biology are becoming exceedingly important.

### Itpkb Dampens SOCE in B Cells

Chemically induced *Itpkb gKO* mice showed overall normal B cell development in the BM but had markedly reduced numbers of all splenic B cell subsets (39, 41). Further studies showed that Itpkb is essential for the selection of functional B cells. To avoid autoimmunity, B cells carrying a self-reactive B cell receptor (BCR) are tolerized through clonal deletion, functional inactivation (anergy), or BCR editing to a different antigen specificity (153). Mature B cells from *Itpkb^−/−^* mice shared many features with B cells from *BCR* and *BCR-antigen transgenic* anergy models (154). Examples are IgM downregulation, impaired BCR-driven proliferation, reduced upregulation of surface-CD69, CD86, and MHCII, and decreased antibody responses to T cell-independent antigens (29, 39, 41). Responses to LPS or CD40 stimulation were normal. In the *HEL BCR transgenic* model, *Itpkb* loss converted responses to mild BCR stimulation from activation to anergy, and responses to moderate stimuli from anergy to deletion (41). This resembles the effect of losing other inhibitors of BCR signaling, such as CD22, SH2 domain containing phosphatase-1, or the Src family protein tyrosine kinase Lyn (8, 153). In developing B cells, Itpkb thus prevents mild BCR stimuli from inducing tolerance and ensures that only B cells expressing self-reactive BCRs are tolerized.

The Schurmans group found overall similar changes in B cell development and impaired T cell-independent antibody responses in *Itpkb gKO* mice. This was associated with reduced *in vitro* survival of *Itpkb^−/−^* B cells, which upregulated pro-apoptotic Bim (29). Bim haploinsufficiency or transgenic expression of anti-apoptotic Bcl-2 increased B cell numbers in *Itpkb^−/−^* mice. Bcl-2 expressing *Itpkb^−/−^* B cells showed diminished BCR-induced Erk activation. The authors used data from non-lymphoid COS cells to suggest that IP_4_ increases B cell survival by sequestering the IP_4_-binding, Ras-inactivating protein RASA3/Gap1^IP4BP^ (155) in the cytosol, resulting in sustained Ras/Erk activation, Bim-phosphorylation, and Bim-degradation (29). However, without confirmation in B cells, the physiological relevance of RASA3/Gap1^IP4BP^ regulation by IP_4_ remains unclear. Later, the same group used *3-83μδ BCR transgenic* mice to explore Itpkb roles in B cell tolerance (42). They found that in a context of mild BCR engagement, *Itpkb* loss impaired B cell maturation and viability, again associated with Bim upregulation. B cell deletion in a context of stronger BCR engagement was unimpaired. Overall, these findings support a shift from B cell functionality or anergy to deletion when Itpkb is lost. Although both BCR-transgenic models revealed surface IgM downregulation on *Itpkb^−/−^* B cells, some differences in the specific response patterns to increasing BCR engagement likely reflect different signaling capacities of the two transgenic BCRs.

In BCR-transgenic anergy models, constitutive expression of self-antigens causes BCR desensitization with defective activation of proximal Lyn/Syk kinases and downstream PLCγ2, IP_3_ production, and Ca^2+^ mobilization (8, 153). By contrast, chemically induced *Itpkb^−/−^* anergic B cells showed overall normal BCR activation of Lyn, Btk, PLCγ2, Erk1/2, and IKKα/β and control-like IP_3_-production, but increased SOCE (39). SOCE normalization by exogenous cell-permeable IP_4_ suggested that the elevated SOCE might result from impaired SOCE dampening by IP_4_ (Figure 5). The Schurman group initially reported reduced BCR- or ionomycin-induced Ca^2+^ influx in *Itpkb^−/−^* B cells (29). However, *3-83μδ BCR transgenic Itpkb^−/−^* B cells showed an elevated BCR-induced Ca^2+^ influx compared to *Itpkb^+/+^* controls, similar to chemically induced *Itpkb^−/−^IgHEL transgenic* and non-BCR transgenic mice (39, 41). The reason for the discrepant Ca^2+^ defects in the original *Itpkb^−/−^* mouse cohort remains unclear, but might include differentially augmented B cell deletion between the models, or effects of an altered Bim/Bcl-2 ratio on IP_3_-receptor function in those particular B cells (42, 156). Consistent with this view, *Itpkb^−/−^IgHEL* transgenic mice showed neither increased negative selection nor Bim accumulation (41). Alternate explanations might include differences in housing, health status, genetic background, or age of the mice used in the different studies.

Despite minor differences, all four studies support a pivotal Itpkb role in dampening BCR signaling to prevent aberrant B cell tolerization. By augmenting BCR signaling, *Itpkb* loss induces anergy of B cells expressing low-to-moderately self-reactive BCRs, but deletion of normally anergic B cells expressing more strongly self-reactive BCRs (41, 42). Thus, Itpkb feedback inhibits BCR signaling to broaden the repertoire of immature B cells that survive negative selection. This positions the BCR selection window appropriately to ensure a normal B cell repertoire that is further tuned through BCR editing. One prediction of this model would be an increased generation of self-reactive B cells which might eventually cause autoimmune disease. Reported diminished BCR light-chain editing in *Itpkb^−/−^* vs. *wild-type* B cells suggests that such autoreactive cells would probably not be “reprogrammed” through receptor editing (157). However, neither *Itpkb^−/−^* mice nor mixed radiation chimeras of *Itpkb^−/−^* BM with *wild-type* T, B, and myeloid cells have shown signs of autoimmunity (8). This could reflect perturbed positive Itpkb functions in peripheral B cells, or the premature death of *Itpkb^−/−^* mice due to HSC defects (26) or infections (47) before autoimmunity can develop. Conditional *Itpkb* disruption in developing vs. mature B cells might prevent some of these problems and help clarify this conundrum, in particular when combined with detailed analyses of the BCR repertoire.

Indeed, a recent study reported that after tamoxifen-induced *Itpkb* deletion in all cells, *Itpkb cKO* mice had near normal B cell numbers and T cell-independent immunization responses associated with reduced Ca^2+^ ER release but elevated SOCE in B cells (59). So, induced *Itpkb* loss recapitulated the SOCE increase in germline *Itpkb^−/−^* B cells but had no major effects on B cell development, homeostasis, viability, and function. Similarly, GNF362 pan-Itpk inhibitor treatment reduced BCR-induced Ca^2+^ ER release but augmented SOCE in wild-type B cells (59). These effects strikingly resemble those reported for *Itpkb^−/−^* neutrophils (23, 112). They also resemble the elevated SOCE in *Itpkb cKO* and GNF362-treated wild-type T cells, although ER release was not detected there (59). Interestingly, GNF362 still inhibited ER release in *Itpkb^−/−^* B cells, but without affecting SOCE (59). So, in murine B cells, SOCE is primarily dampened by Itpkb, but ER release requires additional IP_3_ 3-kinases such as Itpkc, whose loss-of-function in human B cells elevated basal and ionomycin-induced Ca^2+^ levels (44).

The overall normal B cell homeostasis and function in *Itpkb cKO* mice suggest that the increased tolerance of *Itpkb gKO* B cells results from their altered development and selection. The precise functional consequences of *Itpkb* loss in mature B cells remain to be elucidated. Drawing on the phenotypes of EBV-transformed human B cells carrying the *ITPKC^lof^* allele (44), one might expect NLRP3 hyperactivation. It will be interesting to assess if *Itpkb cKO* mice hyperproduce immunoglobulins or pro-inflammatory cytokines and develop inflammatory disease.

It is intriguing that the main molecular defect in *Itpkb^−/−^* and *ITPKC^lof^* B cells is aberrant Ca^2+^ mobilization. While effects on basal Ca^2+^ levels and ER store-release are discrepant (possibly depending on model system and assay conditions), elevated SOCE emerges as a common effect (Figure 5). This suggests that the main function of Itpkb/c and IP_4_ in B cells is to inhibit BCR-induced Ca^2+^ signaling. The precise mechanism causing the elevated SOCE in *Itpkb-* or *Itpkc*-deficient B cells and other immune cells remains unknown. We discuss possibilities in Section “Conclusion and Perspectives.” Beyond elucidating this mechanism, establishing causality of the elevated SOCE for the B cell phenotypes remains important.

Itpkb’s pivotal role in controlling B cell development and function is further emphasized by the recent association of a microdeletion which causes *ITPKB* deficiency in humans with a common variable immunodeficiency (CVID) (158). A patient carrying this microdeletion expressed reduced ITPKB protein. He had reduced serum IgG and IgA, but normal IgM levels and suffered from recurrent skin infections and other symptoms. He did not respond to T cell-independent *Streptococcus pneumoniae* vaccinations and had decreased numbers of T, T_reg_, and NK cells, but normal B cell numbers with increased proportions of marginal zone, transitional, memory, and CD21^low^ B cells. Antigen-induced lymphocyte proliferation and neutrophil oxidative burst were severely impaired. Although additional genes are likely affected by the microdeletion and incomplete ITPKB protein loss, two *ITPKB* missense mutations and a synonymous variant may all explain differences between this patient and the *KO* mice, mechanistic studies to confirm causality of the *ITPKB* mutation for the CVID should prove exciting.

Limiting hematopoietic cell-intrinsic PI3K signaling is critical for preventing blood cancers. In mice, SHIP-1 and PTEN deficiency in B cells caused B cell lymphoma associated with excessive PI3K/Akt signaling (12). Human diffuse large B-cell lymphoma (DLBCL) samples under-expressed PTEN and SHIP-1 (12, 159), and human mantle cell lymphoma samples under-expressed PTEN (160). Reduced PTEN expression or predicted oncogenic PI3Kα mutations associated with poor survival in DLBCL (159) and a third of Burkitt’s lymphomas have inactivating *PTEN* mutations (161). Although no significant changes in PI3K signaling in B cells have been reported in *Itpkb^−/−^* mice, it is attractive to speculate that Itpkb or redundant IP_3_ 3-kinases could have tumor-suppressor functions by dampening PI3K signaling through IP_4_/PIP_3_ antagonism. Consistent with this view, a large-scale retroviral mutagenesis screen identified *Itpkb* as one of the 50 most important common insertion sites in murine lymphoma. *Itpkb* insertions were anti-correlated with insertions in *Pik3cd* encoding PI3Kδ (162, 163). But no blood cancer phenotypes have been reported in *Itpkb^−/−^* mice. As discussed before, this could reflect their premature death due to BM failure, anemia, or infections (26, 47), or partial Itpkb redundancy with Itpka, Itpkc, or IPMK. Conditional *Itpkb* disruption in the B cell lineage to avoid anemia and infections, or breeding *Itpkb^−/−^* mice into blood cancer models will be required to further explore possible Itpkb tumor-suppressor functions. Co-disruption of several *IP_3_3-kinases* can address possible redundancy.

Consistent with an ITPKB tumor-suppressor function in human blood cancers, large-scale whole exome sequencing has identified three different *ITPKB* somatic mutations as candidate CLL drivers in 2% of human patients (164). Two frameshift mutations will remove the Itpkb catalytic domain and thus impair IP_4_ production; the effects of a T_626_S mutation remain to be explored. Several other studies have found *ITPKB* locus deletions, copy number reductions, or missense mutations in patients with DLBCL, Burkitt’s lymphoma, or transformed FL, which often progresses to DLBCL (165–170). Their pathological relevance and underlying mechanisms are unknown. Finally, another retroviral mutagenesis screen found insertions in *Itpkb* to synergize with a retrovirally expressed, AML-associated *Runx1-*mutant in promoting murine BM progenitor outgrowth (171). The same study found that *ITPKB* amplifications and mRNA upregulation associate with poor survival in human AML. However, retroviral insertion can activate or inactivate genes, Itpkb protein levels, function and causality were unassessed and in another study, Itpkb knockdown increased human AML cell expansion (172). Thus, the precise function of Itpkb in AML remains unclear.

### Itpkb Dampens SOCE in Neutrophils

IP_4_ limitation of SOCE may not be limited to lymphocytes. This is suggested by the decreased Ca^2+^ store release but enhanced SOCE in *Itpkb^−/−^* neutrophils (Figure 5) (23), discussed above. Its functional consequences remain to be elucidated.

## Does Itpkb Inhibition have Therapeutic Potential in Human Diseases?

The T and B cell defects in *germline Itpkb^−/−^* mice sparked efforts to develop specific and selective Itpkb small-molecule inhibitors as potential therapeutics for autoimmune disorders or transplant rejection, reviewed in detail in Ref. (8, 149). Consistent with the distinct structural features and biochemical properties of IP_3_ 3-kinases, several different inhibitors have been developed. However, many lack the required potency, isoform selectivity, specificity, and oral bioavailability. Some show high Itpk selectivity over IPMK, but none is exclusively selective for Itpkb (54, 59, 149, 173–175). A possible utility of Itpk inhibitors for immunosuppression is also supported by the T cell impaired phenotypes of *Itpkb cKO* mice and mice treated with the oral pan-Itpk inhibitor GNF362, and by the GNF362 efficacy in a rat antigen-induced arthritis model (59). Interestingly, induced *Itpkb* deletion in adult mice, or GNF362 treatment of adults unveiled no major defects in B cell function. Thus, the efficacy of any treatment of adults with Itpk inhibitors might primarily rely on T cell inhibition, limiting the utility of this approach to T cell-mediated diseases. A therapeutic Itpk inhibitor would need to be exquisitely selective for Itpkb to avoid inhibition of Itpkc, whose lof hyperactivates T cells, B cells, and macrophages and has been implicated in human inflammatory KD, Hirschsprung disease, calcium nephrolithiasis, and cervical squamous cell carcinoma (150–152). GNF362 does inhibit Itpka and Itpkc (59), but the relevance of their co-inhibition for any phenotypes remains to be elucidated. Any therapeutic approach would also need to avoid the CVID, BM failure/anemia, and possibly neutrophil hyperactivity found in human patients or mice with persistent *Itpkb* lof (22, 23, 26, 57, 158), and the disruption of possible Itpkb tumor-suppressor functions discussed in the B cell section. Based on the common reversibility of drug-induced HSC mobilization (85–87) and the dependence of many neutrophil functions on B cell-produced immunoglobulins (22, 23, 57), transient or intermittent Itpkb inhibition might mitigate some of these liabilities and might possibly even be able to expand HSC for therapeutic engraftment (26, 30). Finally, further elucidation of potential Itpkb roles in Alzheimer’s disease (176, 177), multiple sclerosis (178), and malignant melanoma (179) might unveil additional therapeutic opportunities or liabilities for selective Itpkb inhibitors. It will be particularly interesting to study whether Itpkb-dependent immunological mechanisms contribute to these diseases.

## Conclusion and Perspectives

The data reviewed above have identified Itpkb, Itpkc, and IP_4_ as critical regulators of the development and function of most hematopoietic and immune cell types (Figure 3). IP_4_ primarily acts through two mechanisms: non-canonical PIP_3_ antagonism to dampen PI3K signaling, and SOCE dampening to restrict Ca^2+^ mobilization. PIP_3_ antagonism has been relatively well established, but one remaining puzzle discussed above is why PI3K signaling appears normal in *Itpkb^−/−^* B cells. The precise molecular mechanism through which Itpks and IP_4_ inhibit SOCE, however, remains to be determined, and a formal proof that elevated SOCE causes the associated B cell, T cell, and neutrophil phenotypes is lacking. SOCE dampening might possibly include IP_4_-blockade of the polybasic region in STIM1 which mediates plasma membrane recruitment, IP_3_-turnover by Itpks, other controversial IP_3_ 3-kinase or IP_4_-roles in Ca^2+^-mobilization, or other functions of IP_4_ or its metabolites (8, 45, 119).

SOCE-modulation, additional unknown mechanisms of Itpkb/c and IP_4_ action, or partial redundancy of Itpka-c and IPMK could explain some of the phenotypic discrepancies between mice or humans lacking Itpkb, Itpkc, SHIP, or PTEN, reviewed above for each affected cell type. Discussed in detail elsewhere (8, 19, 38, 45), additional relevant mechanisms might involve other lymphocyte-expressed IP_4_-binding proteins beyond Tec kinases and Akt, including PDK1 (180), RASA2/GAP1^m^, RASA3/GAP1^IP4BP^, centaurin-α1, cytohesins, or synaptotagmins. Indeed, impaired RASA3 sequestration from the plasma membrane by IP_4_ has been suggested to cause Ras/Erk hyperactivation in *Itpkb^−/−^*-deficient thymocytes and B cells, although whether this occurs in lymphocytes and is physiologically relevant remains to be shown (28, 29). Identifying the entire complement of IP_4_-binding proteins in hematopoietic cells, and delineating their functions, will be important for a more comprehensive elucidation of how this pivotal soluble messenger controls hematopoiesis. In particular, it will be interesting to explore why Itpkc may have distinct functions in murine vs. human T cells, and what determines which mechanisms Itpks or IP_4_ engage in a given cell type, and whether they promote (as in DP thymocytes) or dampen (as in DN thymocytes, peripheral T, B, and NK cells, HSC, GMP, monocytes/macrophages, and neutrophils) immunoreceptor signaling and immune cell function.

Possible explanations could include the Orai-mediated SOCE requirement in mature immunocytes but not thymocytes (119), or differing Itpk or IP_4_ functions in different cellular or signaling contexts, or after different intensities of the input signal (8). Itpkb controls SOCE in T cells but not DP thymocytes, and Tec kinases in DP thymocytes but not B cells (8, 25, 26, 45). Moreover, thymocyte positive selection is triggered by mild and/or transient TCR signals in DP thymocytes and requires IP_4_. Negative selection is mediated by strong and/or sustained TCR signals and might be less impaired in *Itpkb^−/−^* mice (20, 47, 181). Peripheral T cells also generate strong TCR signals that might be impacted differently by IP_4_ deficiency. Our mathematical modeling studies suggested that a combination of IP_4_ positive (at low concentrations) and negative (at high concentrations) feedback would make TCR signaling most robust (21). Thus, a re-evaluation of Itpk functions in immunoreceptor signaling circuitries from a systems-perspective might prove informative. Alternatively, the effects of Itpks and IP_4_ might depend on their cellular concentration, subcellular localization, posttranslational modification, or on the specific IP_4_ effectors or metabolites present in a cell. Distinct roles of different Itpks could also involve IP_4_-unrelated noncatalytic functions of Itpka/b but not Itpkc in actin bundling (8, 45, 144, 182).

Possible contributions of IP_4_ metabolites are illustrated by the role of IP_7_ in dampening PIP_3_ function in neutrophils (24). Moreover, a recent study unveiled its precursor inositol-hexakisphosphate (IP_6_) as a candidate regulator of the B cell expressed Tec-kinase Btk (183). *In vitro*, physiological IP_6_ concentrations activated Btk by binding to a specific site in its PH–Tec-homology (TH) domain unrelated to its PIP_3_-binding site. IP_6_ sandwiching between two PH–TH domains might enable transient Btk dimerization and activation. While the physiological relevance of this mechanism remains to be shown, it might provide a second example beyond IP_4_ regulation of Itk for how soluble IPs could promote PH domain function. Interestingly, both examples involve PH domain oligomerization (20, 183). Among the ~234 mammalian PH domains, only ~10% bind phosphoinositides, and only those of Itk and perhaps dynamin have been shown to oligomerize in cells (1). If PH domain oligomerization is required for their positive regulation by IPs, this mode of regulation might thus be rare. But then, soluble IP_4_ might promote PIP_3_ binding of Tec and RASA3, whose PH domain oligomerization status remains unknown (20). Thus, elucidating what determines whether an IP promotes or inhibits the function of a given PH domain, or does not affect it at all, remains an important future direction.

Beyond acting as protein ligands, inositol-pyrophosphates including IP_7_ can also act as non-enzymatic protein-phosphorylating agents (113–115). Whether this controls hematopoietic cell functions remains to be elucidated. Clearly, deciphering the functions of unstudied “inositol code” members in hematopoietic cells promises to open up exciting and unexpected novel biology (8, 56).

Given the paramount importance of PIP_3_ regulation through its turnover by SHIP and PTEN (10–12), one wonders whether IP_4_ and IP_7_ might also be controlled via turnover. *In vitro*, several phosphatases including SHIP-1/2 can dephosphorylate the 5-positions of IP_3_ and IP_4_, and PTEN can convert IP_4_ into IP_3_ (184–188). Whether this occurs *in vivo* is unknown, although Jurkat T cells contain an unknown IP_4_ 5-phosphatase unrelated to SHIP-1 (60, 189). *In vivo* studies of IP_4_ turnover appear worthwhile.

Except for one recent study focused on peripheral T and B cells (59), most of the published data about *in vivo* IP_3_ 3-kinase functions to date were obtained in germline knockout mice. The B cell tolerance in mice with constitutive but not acutely induced Itpkb inactivation (59) illustrates that some of the germline knockout phenotypes likely include secondary effects of earlier defects in hematopoiesis, or sustained extrinsic effects of *Itpkb* loss in other cell types. It will therefore be important to confirm developmental stage-specific cell-intrinsic *Itpkb/c* and IP_4_ functions in appropriate conditional knockout mice. Concluding, Itpks and IP_4_ clearly play exciting and important roles in hematopoietic cells, but much work remains to be done to fully elucidate the roles of the “inositol code.” We can expect fascinating results.

## Author Contributions

ME and KS wrote this review, prepared figures and revised this review.

## Conflict of Interest Statement

KS is an employee of Pfizer, Inc. The remaining coauthor declares that the research was conducted in the absence of any commercial or financial relationships that could be construed as a potential conflict of interest.
